# Tim-3 regulates the immunosuppressive function of decidual MDSCs via the Fyn-STAT3-C/EBPβ pathway during *Toxoplasma gondii* infection

**DOI:** 10.1371/journal.ppat.1011329

**Published:** 2023-04-14

**Authors:** Houbao Qi, Yuantao Li, Xianbing Liu, Yuzhu Jiang, Zhidan Li, Xiaoyan Xu, Haixia Zhang, Xuemei Hu

**Affiliations:** 1 Department of Immunology, Binzhou Medical University, Yantai, Shandong, P. R. China; 2 Department of Gynecology and Obstetrics, Yantai Affiliated Hospital of Binzhou Medical University, Yantai, Shandong, P. R. China; University of Wisconsin Medical School, UNITED STATES

## Abstract

Myeloid-derived suppressor cells (MDSCs) play a key role in maintaining maternal-fetal tolerance for a successful pregnancy, but the role of MDSCs in abnormal pregnancy caused by *Toxoplasma gondii* infection is unknown. Herein, we revealed a distinct mechanism by which T-cell immunoglobulin domain and mucin domain containing protein-3 (Tim-3), an immune checkpoint receptor that balances maternal-fetal tolerance during pregnancy, contributes to the immunosuppressive function of MDSCs during *T*. *gondii* infection. The expression of Tim-3 in decidual MDSCs was significantly downregulated following *T*. *gondii* infection. The proportion of monocytic MDSCs population, the inhibitory effect of MDSCs on T-cell proliferation, the levels of STAT3 phosphorylation, and the expression of functional molecules (Arg-1 and IL-10) in MDSCs were all decreased in *T*. *gondii-*infected pregnant Tim-3 gene knockout (Tim-3KO) mice compared with infected pregnant WT mice. After treatment with Tim-3-neutralizing Ab *in vitro*, the expression levels of Arg-1, IL-10, C/EBPβ, and p-STAT3 were decreased, the interaction between Fyn and Tim-3 or between Fyn and STAT3 was weakened, and the binding ability of C/EBPβ to the promoters of *ARG1* and *IL10* was decreased in human decidual MDSCs with *T*. *gondii* infection, while opposite results were observed following treatment with galectin-9 (a ligand for Tim-3). Inhibitors of Fyn and STAT3 also downregulated the expression of Arg-1 and IL-10 in decidual MDSCs and exacerbated adverse pregnancy outcomes caused by *T*. *gondii* infection in mice. Therefore, our studies discovered that the decrease of Tim-3 after *T*. *gondii* infection could downregulate the functional molecules of Arg-1 and IL-10 expression in decidual MDSCs through the Fyn-STAT3-C/EBPβ signaling pathway and weaken their immunosuppressive function, which eventually contribute to the development of adverse pregnancy outcomes.

## Author summary

Pregnant women in their first trimester with a primary infection of *T*. *gondii* are particularly susceptible resulting in a number of serious adverse pregnancy outcomes. Previous studies have shown that *T*. *gondii* infection results in the dysfunction of immune cell function at the maternal-fetal interface, which destroys the function of maternal-fetal immune tolerance for a successful pregnancy. MDSCs have an immunosuppressive capacity and play an important role in the maintenance of normal pregnancy. However, the role of MDSCs in the adverse pregnancy outcomes caused by *T*. *gondii* infection remains unknown. Here, we analyzed the effect of an immunosuppressive molecule Tim-3 on the function of decidual MDSCs following *T*. *gondii* infection. Our results showed that *T*. *gondii* infection downregulated the proportion of monocytic MDSCs population, the inhibitory ability of MDSCs on T cell proliferation, the level of STAT3 phosphorylation, and the expression of functional molecules (Arg-1 and IL-10) in MDSCs from the pregnant Tim-3KO mice compared with the pregnant WT mice. Further study showed that the decrease of Tim-3 expression caused by *T*. *gondii* infection could downregulate Arg-1 and IL-10 expression in decidual MDSCs via the Fyn-STAT3-C/EBPβ signaling pathway and weaken their immunosuppressive function. This work suggested that MDSCs dysfunction resulting from the downregulation of Tim-3 after *T*. *gondii* infection may be an important mechanism of adverse pregnancy outcomes, which may provide theoretical information for prophylactic and/or therapeutic treatments against the infection-related abnormal pregnancy.

## Introduction

*Toxoplasma gondii* is an obligate intracellular parasite that causes human TORCH syndrome and can result in serious abnormal pregnancy outcomes such as abortion, preterm birth, and stillbirth in pregnant women with a primary infection [1]. Several studies have shown that *T*. *gondii* infection during pregnancy leads to the dysfunction of decidual immune cells and the aberrant expression of immune regulatory molecules at the maternal-fetal interface, which may be the molecular immunologic mechanisms by which *T*. *gondii* causes abnormal pregnancy outcomes [2–4]. However, the exact pathogenic mechanism remains largely unclear.

During pregnancy, many immune cells, such as natural killer (NK) cells, macrophages, myeloid-derived suppressor cells (MDSCs), dendritic cells (DCs), and regulatory T (Treg) cells, exist in the maternal-fetal interface and have been reported to play important roles in maintaining maternal-fetal immune tolerance for a successful pregnancy [1, 2]. MDSCs are a group of heterogeneous immature cells derived from the bone marrow that suppress immune responses through multiple mechanisms. Murine MDSCs, characterized by the expression of Gr-1 and CD11b, can be mainly divided into two major subsets: monocytic MDSCs (M-MDSCs, which can be identified as CD11b^+^Gr-1^low^ or CD11b^+^Ly6G^-^Ly6C^hi^) and granulocytic MDSCs (G-MDSCs, which can be identified as CD11b^+^Gr-1^hi^ or CD11b^+^Ly6G^+^Ly6C^low^) [5]. Human MDSCs are identified as CD11b^+^CD33^+^HLA-DR^neg/low^ cells [6]. These cells have potent suppressive activity in both innate and adaptive immune responses.

The immunosuppressive capacity of MDSCs is associated with many functional molecules, including arginase-1 (Arg-1), inducible nitric oxide synthase (iNOS), indoleamine 2,3-dioxygenase (IDO), interleukin-10 (IL-10), programmed death ligand 1 (PD-L1), and Tim-3 [7]. Studies have revealed that MDSCs are activated in a variety of pathological conditions, such as infection, chronic inflammation, cancer, and autoimmune diseases [8, 9]. Combined with their immunosuppressive effects, MDSCs accumulate in the decidua during pregnancy and play a key role in maintaining maternal-fetal tolerance [10]. MDSCs can efficiently suppress T-cell proliferation by expressing Arg-1, iNOS, and IL-10 in the decidua to maintain maternal-fetal tolerance [11]. In addition, MDSCs enhance the suppressive activity of decidual NK cells and Treg cells through the production of cytokines such as IL-10 [11]. The absence or dysfunction of MDSCs has been found in both humans and mice with spontaneous abortion, preeclampsia, and intrauterine growth retardation [12–14]. However, the role of MDSCs in the adverse pregnancy outcomes caused by *T*. *gondii* infection remains unknown.

Tim-3 is a type I transmembrane protein that was initially identified as a Th1-specific cell surface molecule that decreases Th1 responses by transducing apoptotic signaling [15]. A previous report showed that Tim-3 with its ligand, galectin-9 (Gal-9), could promote maternal-fetal immune tolerance and reduce pregnancy loss [16]. Our previous studies have indicated that Tim-3 is a vital immune molecule for sustaining the tolerance of decidual macrophages and decidual NK cells and plays an indispensable role in the adverse pregnancy outcomes caused by *T*. *gondii* infection [1, 17]. In addition, Tim-3 is constitutively expressed in MDSCs and regulates the immunosuppressive functions of MDSCs during pregnancy [18]. However, the mechanism by which Tim-3 regulates MDSCs function during adverse pregnancy outcomes caused by *T*. *gondii* infection has not yet been investigated.

In the present study, we found that in *T*. *gondii-*infected Tim-3KO mice, MDSCs exhibited decreased expression of Arg-1 and IL-10 and weakened suppression of CD4^+^ and CD8^+^ T-cell proliferation. These functional changes were correlated with decreases in the signal transducers and activators of transcription 3 (STAT3) and CCAAT enhancer binding protein beta (C/EBPβ) signaling pathways. Furthermore, reduced binding activity of tyrosine-protein kinase Fyn (Fyn) to Tim-3 and to STAT3 was found in *T*. *gondii*-infected human decidual MDSCs treated with Tim-3-neutralizing Ab (αTim-3). Our results demonstrated that Tim-3 could regulate the expression of the functional molecules Arg-1 and IL-10 in decidual MDSCs through the Fyn-STAT3-C/EBPβ signaling pathway, by which *T*. *gondii* infection impairs the immunosuppressive function of MDSCs and eventually contributes to the occurrence of adverse pregnancy outcomes.

## Results

### Tim-3 regulates murine MDSCs expansion and activation during *T*. *gondii* infection

**Fig 1:**
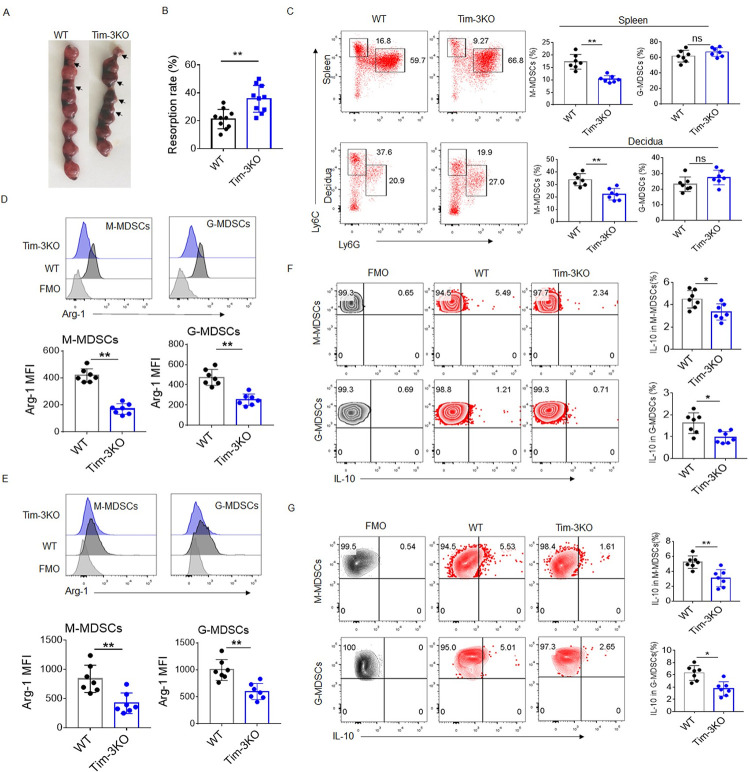
Weakened MDSCs activation in infected pregnant Tim-3KO mice. **(A)** Representative picture of uteri from *T*. *gondii*-infected pregnant wild-type (WT) and Tim-3 gene knockout (Tim-3KO) mice at Gd 12.5. Black arrows indicated resorption sites. **(B)** Scatter diagram with bars showing the resorption rates of the *T*. *gondii*-infected WT and Tim-3KO mice. Resorption rates were defined as the ratio between resorbing units and total implantation sites. Each symbol represents an individual animal, and the median is indicated (n = 10). **(C)** Representative flow cytometry results and statistical analysis of M-MDSCs and G-MDSCs in spleens and deciduae from the infected WT and Tim-3KO mice (n = 7). **(D–E)** Representative flow cytometry results and statistical analysis of Arg-1 expression in M-MDSCs and G-MDSCs from the spleens (D) and deciduae € of the infected WT and Tim-3KO mice (n = 7). **(F–G)** Representative flow cytometry results and statistical analysis of the intracellular level of IL-10 in M-MDSCs and G-MDSCs from the spleens (F) and deciduae (G) of the infected WT and Tim-3KO mice (n = 7). The data are presented as the mean ± SD, *p < 0.05, **p < 0.01, Student’s *t* test.

MDSCs are closely related to maternal-fetal immune tolerance and successful pregnancy [10]. As a negative immune regulator at the maternal-fetal interface, Tim-3 plays a critical role in preventing adverse pregnancy outcomes caused by *T*. *gondii* infection [17]. Therefore, we decided to investigate whether Tim-3 signaling regulates the function of MDSCs during pregnancy with *T*. *gondii* infection. To this end, we explored the characteristics of MDSCs from the Tim-3KO and wild-type (WT) mice with or without *T*. *gondii* infection. Consistent with our previous results [1], there were more severe adverse pregnancy outcomes in the infected Tim-3KO mice (Fig 1A and 1B). In addition, IFN-γ and TNF-α levels in serum, IFN-γ level in decidua were significantly higher in the infected Tim-3KO pregnancy mice than those in the infected WT pregnancy mice (S1A and S1B Fig). On the contrary, the number of parasites in spleen and decidua was decreased in the infected Tim-3KO pregnancy mice compared with the infected WT pregnancy mice (S1C Fig). These results indicated that Tim-3 deficiency might weaken the immune tolerance function and enhance killing against parasite. However, without *T*. *gondii* infection, there was no significant difference in pregnancy outcomes and pro-inflammatory cytokines (IFN-γ and TNF-α) levels in serum and decidua between the WT and Tim-3KO mice (S2A Fig). Interestingly, there was a markedly decreased proportion of CD11b^+^Ly6C^hi^Ly6G^-^ M-MDSCs in the spleen, decidua, and blood from the infected Tim-3KO mice compared to the control infected WT mice shown by flow cytometry (Figs 1C and S3A) on the basis of the gating strategies (S4A Fig). The proportions of MDSCs in the spleen, blood, and decidua in the uninfected Tim-3KO mice were similar to those in the uninfected WT mice (S2B Fig). These results suggested that Tim-3 affects the expansion of MDSCs, which could contribute to the adverse pregnancy outcomes caused by *T*. *gondii* infection.

The immunomodulatory function of MDSCs is dependent on a variety of functional molecules, such as Arg-1 and IL-10 [19]. Flow cytometry revealed lower expression of Arg-1 in MDSCs from the spleen, decidua, and blood of the infected Tim-3KO mice (Figs 1D–1E and S3B). Furthermore, decreased IL-10^+^ M-MDSCs and IL-10^+^ G-MDSCs were found in the spleen, decidua, and blood from the infected Tim-3KO mice compared with those from the infected WT mice (Figs 1F–1G and S3C). Our data indicated that Tim-3 could affect the immunosuppressive function of MDSCs by regulating Arg-1 and IL-10 expression.

### Tim-3 promotes the immunosuppressive function of murine MDSCs during *T*. *gondii* infection

**Fig 2:**
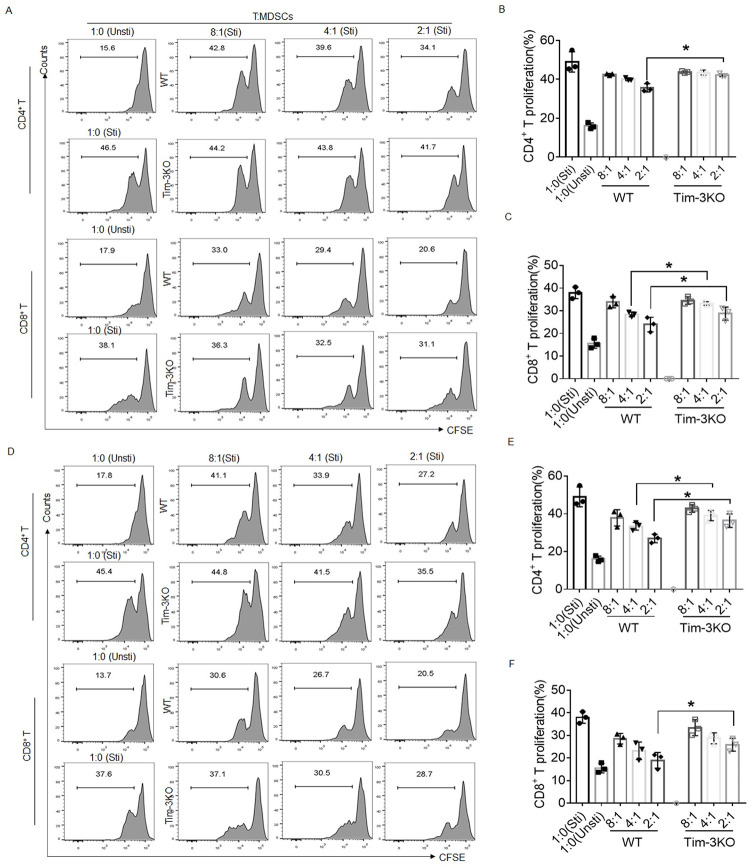
Tim-3 deficiency inhibits the immunosuppressive function of murine MDSCs during *T*. *gondii* infection. **(A–C)** Representative flow cytometry analysis and statistical analysis of the proliferation rates of CD4^+^ T cells (B) and CD8^+^ T cells (C) from WT mice cocultured with MDSCs from the spleens of the infected WT and Tim-3KO pregnancy mice (three independent experiments). **(D–F)** Representative flow cytometry analysis and statistical analysis of the proliferation rates of CD4^+^ T cel€(E) and CD8^+^ T cells (F) from WT mice cocultured with MDSCs isolated from the deciduae of the infected WT and Tim-3KO pregnancy mice (three independent experiments). The data are presented as the mean ± SD, One-way ANOVA, *p < 0.05, **p < 0.01.

To further explore the effect of Tim-3 deficiency on the immunosuppressive ability of MDSCs, we cocultured CD3^+^ T cells from WT mice with MDSCs freshly isolated from the spleens or deciduae of the infected WT and infected Tim-3KO pregnancy mice at a ratio of 1:2, 1:4 or 1:8 in the presence of anti-CD3/CD28 antibody stimulation for 3 days. Splenic MDSCs isolated from the infected Tim-3KO mice had a weakened capacity to suppress the proliferation of CD4^+^ or CD8^+^ T cells (Fig 2A–2C). MDSCs freshly isolated from the deciduae of the infected Tim-3KO mice also exhibited reduced suppression of CD4^+^ and CD8^+^ T-cell proliferation, indicating that Tim-3 knockout inhibits the T-cell suppressive capacity of MDSCs following *T*. *gondii* infection (Fig 2D–2F). Thus, our data indicated that Tim-3 signaling may contribute to maintaining the immunosuppressive function of murine decidual MDSCs during *T*. *gondii* infection.

### Tim-3 regulates human decidual MDSCs activation after *T*. *gondii* infection

**Fig 3:**
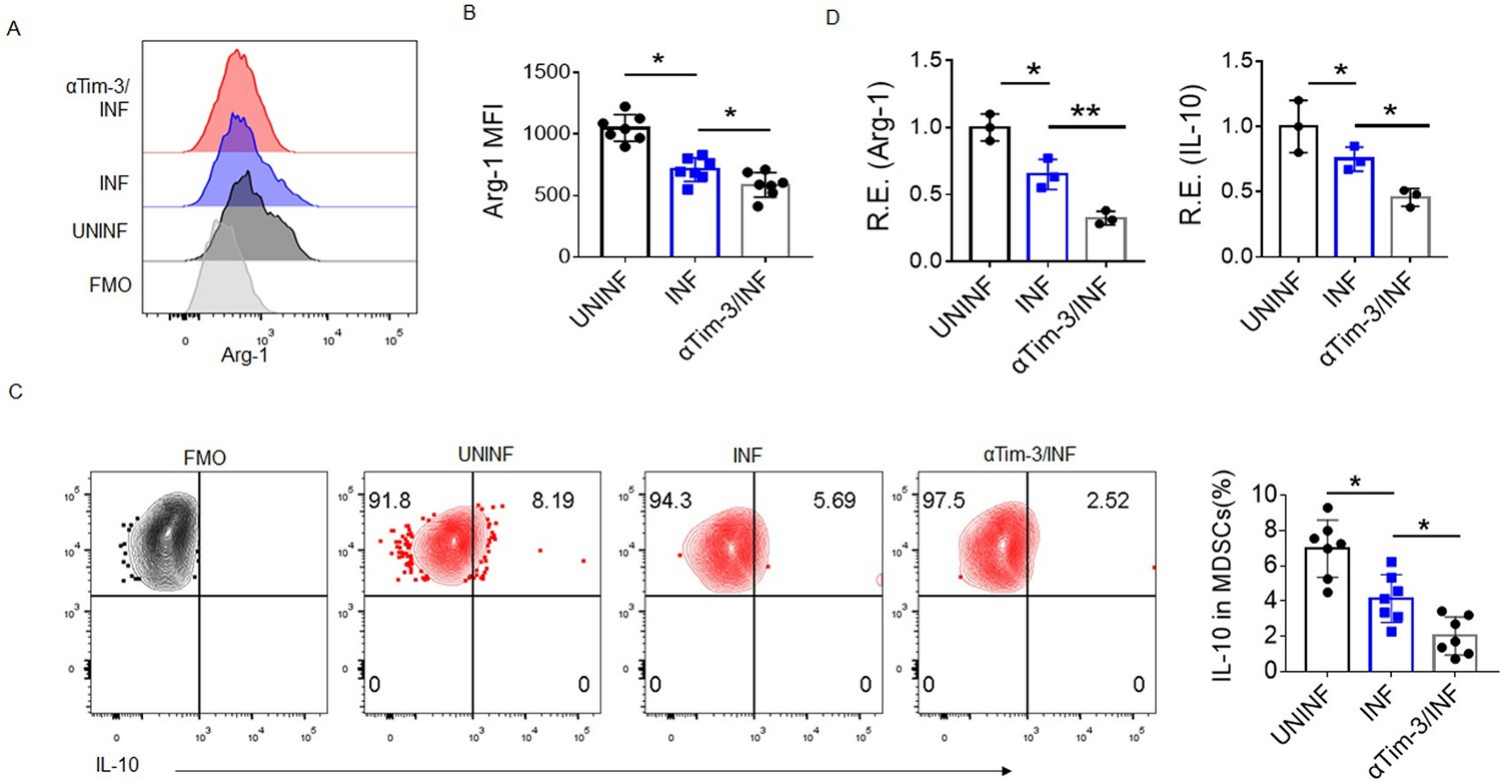
The blockage of Tim-3 by a neutralizing antibody impedes human decidual MDSCs activation during *T*. *gondii* infection. **(A–B)** Representative flow cytometry results (A) and statistical analysis (B) of Arg-1 expression in human decidual MDSCs from groups of normal control (UNINF), *T*. *gondii* infection (INF), and Tim-3 neutralizing antibody (αTim-3) treatment plus *T*. *gondii* infection (αTim-3/INF) (data represent the mean ± SD from 7 human samples). **(C)** Representative flow cytometry results and statistical analysis of the intracellular level of IL-10 in human decidual MDSCs in the UNINF, INF, and αTim-3/INF groups (data represent the mean ± SDs from 7 human samples). Cells from UNINF group were used for the FMO conditions. **(D)** qPCR was used to analyze the expression of Arg-1 and IL-10 in the infected human decidual MDSCs after αTim-3 treatment (data represent the mean ± SD from three independent experiments). *p < 0.05, **p < 0.01, One-way ANOVA.

To investigate the regulatory effect of Tim-3 signaling pathway on decidual MDSCs, mononuclear cells were isolated from human decidual tissues treated with *T*. *gondii* infection and/or αTim-3. In the condition of *T*. *gondii* infection, we found that the expression of Arg-1 was obviously downregulated in the human decidual MDSCs after the treatment with αTim-3 (Fig 3A and 3B) using flow cytometry based on the gating strategies (S4C Fig), while αTim-3 had no significantly effect on Arg-1expression in human decidual MDSCs without *T*. *gondii* infection (S5 Fig). Flow cytometry also revealed a decreased proportion of IL-10^+^ MDSCs after αTim-3 treatment (Fig 3C). In addition, significantly lower expression of Arg-1 and IL-10 was found by qPCR in isolated human decidual MDSCs after *T*. *gondii* infection, and the expression levels were further decreased after αTim-3 treatment (Fig 3D). These data indicated that Tim-3 enhances the expression of Arg-1 and IL-10 in human decidual MDSCs, which is closely related to MDSCs activation.

### Tim-3 regulates the expression of Arg-1 and IL-10 in MDSCs through the Fyn-STAT3-C/EBPβ pathway

**Fig 4:**
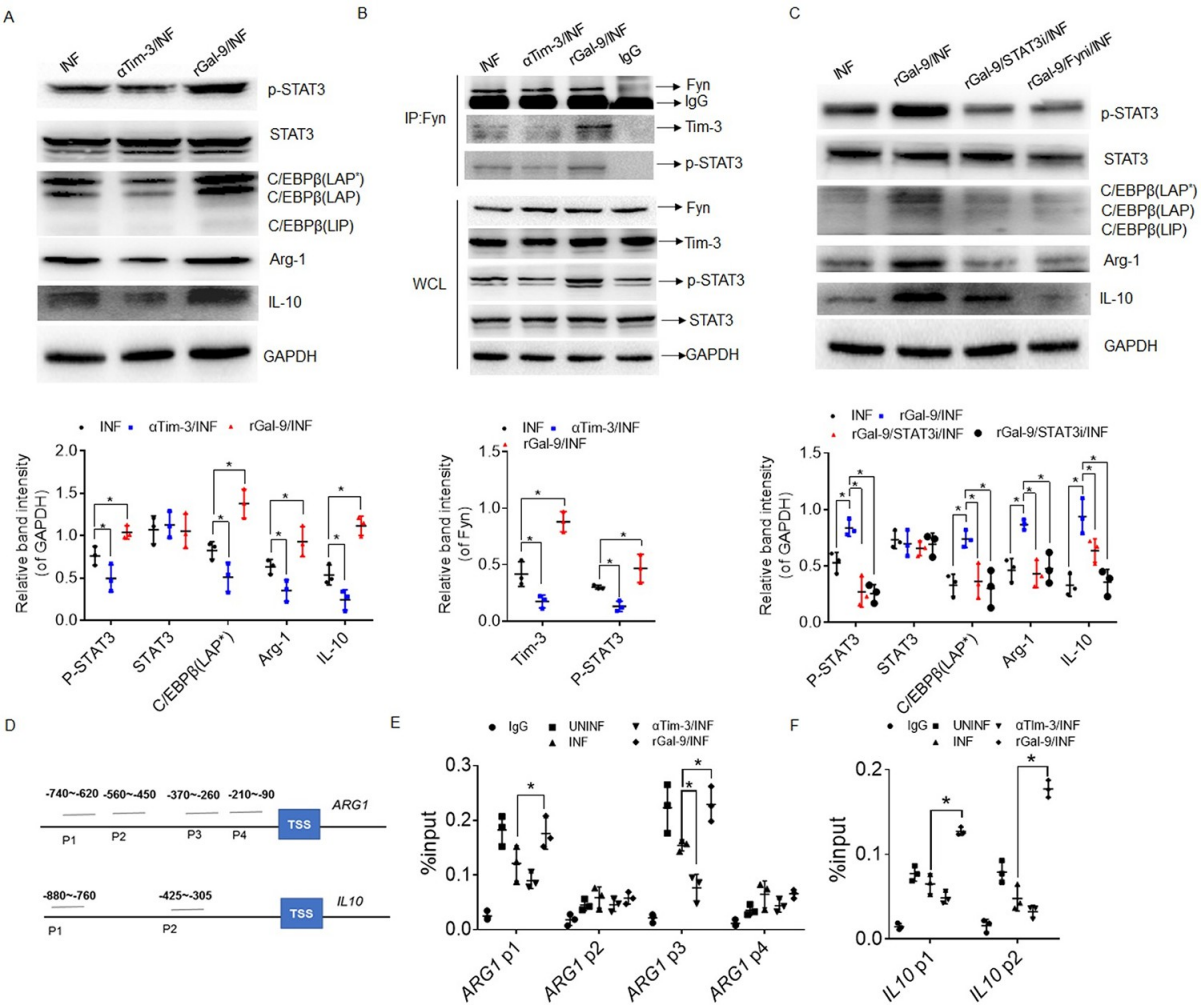
The Tim-3-Fyn-STAT3-C/EBPβ pathway mediates Arg-1 and IL-10 expression in MDSCs following *T*. *gondii* infection. **(A)** Western blot analysis and statistical analysis of the expression of STAT3, phosphorylated (p)-STAT3, C/EBPβ, Arg-1, and IL-10 in MDSCs treated with αTim-3 or artificial recombinant galectin-9 (rGal-9) during *T*. *gondii* infection. The quantified relative band intensity was determined by ImageJ (similarly hereinafter). The results are shown as the mean ±SD of three separate experiments, One-way ANOVA, *p < 0.05. **(B)** Coimmunoprecipitation (Co-IP) analysis of the interaction between Fyn and Tim-3 and between Fyn and p-STAT3 in the MDSCs treated with αTim-3 or rGal-9 during *T*. *gondii* infection. The results are shown as the mean ±SD of three separate experiments, One-way ANOVA, *p < 0.05. **(C)** Western blot analysis and statistical analysis of the expression of STAT3, p-STAT3, C/EBPβ, Arg-1, and IL-10 in the MDSCs treated with an inhibitor of STAT3 or Fyn in the presence of rGal-9 during *T*. *gondii* infection. The results are shown as the mean ±SD of three separate experiments, One-way ANOVA, *p < 0.05. **(D)** Schematic diagram shows the possible binding site of C/EBPβ on the promoter regions of *ARG1* and *IL10*. **(E–F)** ChIP-PCR of C/EBPβ binding regions on the promoter of€*G1* (E) and *IL10* (F). ChIP assays were performed using anti-C/EBPβ antibody and then qRT‒PCR. The data in all panels are representative of at least three independent experiments. The data are presented as the mean ± SD, *p < 0.05, One-way ANOVA. UNINF, normal control; INF, *T*. *gondii* infection.

We next determined how Tim-3 signaling was formed to regulate the expression of Arg-1 and IL-10 in MDSCs during *T*. *gondii* infection. Previous studies have shown that STAT3 and C/EBPβ are critical transcription factors in the regulation of MDSCs expansion and activation [20]. Thus, we hypothesized that Tim-3 signaling might affect STAT3 and/or C/EBPβ activity to regulate the expression of Arg-1 and IL-10 in MDSCs during *T*. *gondii* infection. Indeed, there was a markedly decreased level of phosphorylated STAT3 (p-STAT3) in the human decidual MDSCs treated with αTim-3 following *T*. *gondii* infection (S7A Fig). Lower expression of p-STAT3 in decidual MDSCs was also found in the infected Tim-3KO mice (S7B Fig). Next, we used an artificial recombinant Tim-3 ligand, galectin-9 (rGal-9), to determine whether Tim-3 signaling regulates STAT3 and/or C/EBPβ activity in decidual MDSCs. Interestingly, the expression levels of p-STAT3, C/EBPβ, Arg-1, and IL-10 were all increased after rGal-9 stimulation with *T*. *gondii* infection and decreased after αTim-3 treatment (Fig 4A), indicating that Tim-3 signaling promotes the activation of STAT3 and the expression of C/EBPβ, which might regulate the expression of Arg-1 and IL-10. Moreover, JSI-124, a selective STAT3 inhibitor, effectively inhibited the expression of C/EBPβ, Arg-1, and IL-10 in the presence of rGal-9 (Fig 4C), implying that Tim-3 signaling regulates the expression of C/EBPβ, Arg-1, and IL-10 by activating STAT3.

Next, how Tim-3 signaling activates STAT3 was investigated. Studies have shown that the Src family kinase Fyn acts as an adaptor protein that binds Tim-3 and downstream signaling molecules [21]. Coimmunoprecipitation assays confirmed that the interactions between Fyn and Tim-3 and between Fyn and p-STAT3 were significantly enhanced by rGal-9 stimulation but weakened under αTim-3 treatment (Fig 4B). Moreover, the Fyn inhibitor saracatinib downregulated the expression of STAT3, C/EBPβ, Arg-1, and IL-10 in the presence of rGal-9 (Fig 4C). These results demonstrated that Tim-3 could activate the Fyn-STAT3-C/EBPβ signaling pathway to regulate the expression of Arg-1 and IL-10 during *T*. *gondii* infection.

C/EBPβ was reported as a transcription factor regulating the expression of Arg-1 and IL-10 [22]. Thus, the activity of C/EBPβ binding to the promotor of *ARG1* and *IL10* was also explored using chromatin immunoprecipitation and qPCR (CHIP‒qPCR) assays. ChIP‒qPCR showed that rGal-9 significantly increased the enrichment of C/EBPβ in the promotor regions of *ARG1*, which span from -740 to -620 bp and from -370 to -260 bp, whereas αTim-3 treatment reduced the enrichment of C/EBPβ in these promoter regions (Fig 4D–4E). In addition, rGal-9 significantly increased the enrichment of C/EBPβ in the -880 to -760 and -425 to -305 regions of *IL10* (Fig 4D and 4F), indicating that Tim-3 signaling enhances the activity of C/EBPβ regulating the expression of *ARG1* and *IL10* in decidual MDSCs with *T*. *gondii* infection. Thus, we concluded that Tim-3 promotes the expression of Arg-1 and IL-10 in MDSCs through the Fyn-STAT3-C/EBPβ pathway during *T*. *gondii* infection.

### JSI-124 weakens decidual MDSCs activation *in vivo* and exacerbates adverse pregnancy outcomes in mice caused by *T*. *gondii* infection

**Fig 5:**
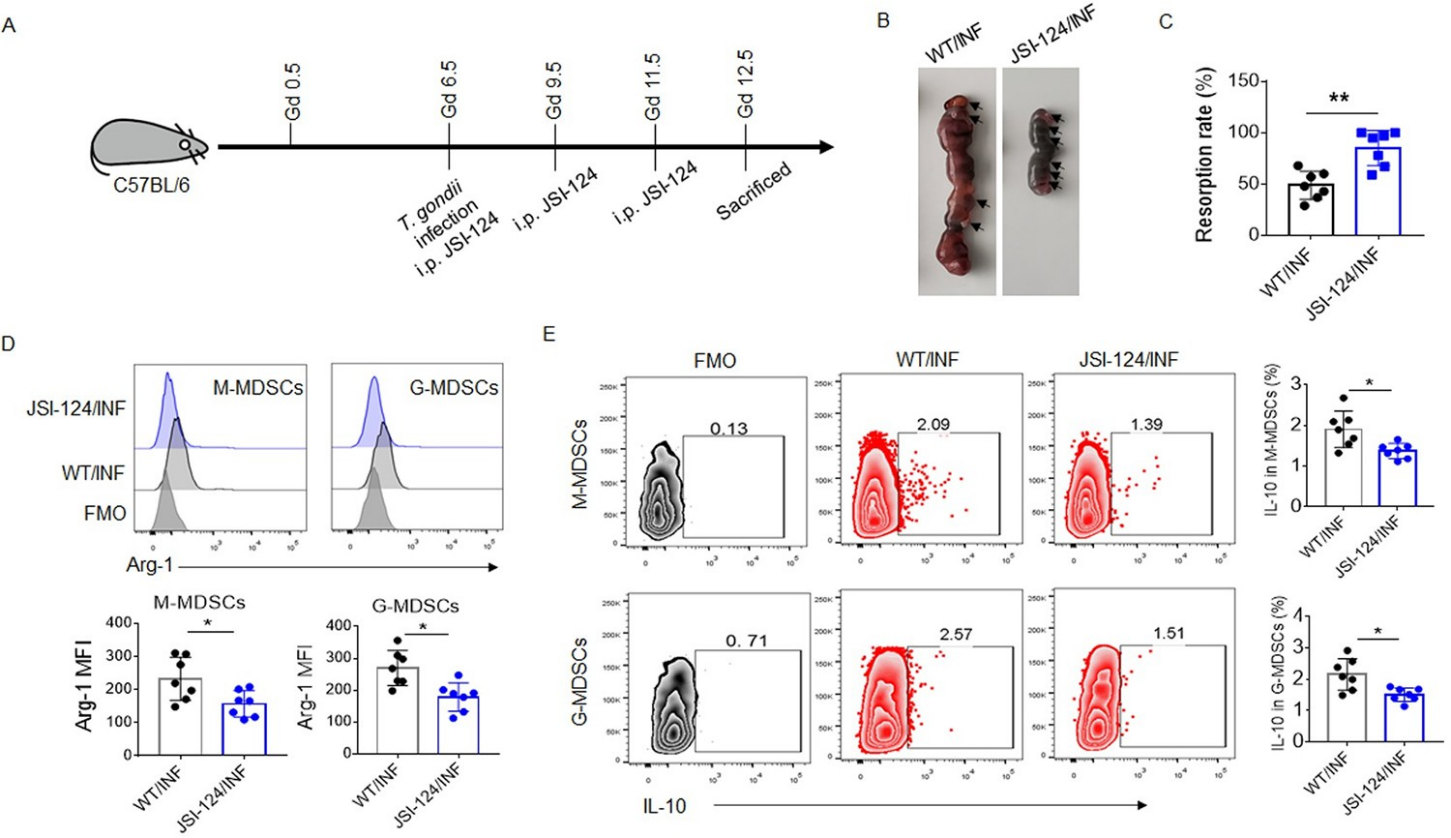
JSI-124 inhibits decidual MDSCs activation during *T*. *gondii* infection *in vivo*. **(A)** Schematic of the experiment. The detailed protocol is described in the *Materials and Methods*. **(B–C)** Representative picture and statistical analysis of uteri from the *T*. *gondii*-infected pregnant WT mice treated with (INF/JSI-124) or without (WT/INF) JSI-124. Black arrows indicated resorption sites. Each symbol represents an individual animal, and the median is indicated (n = 7). **(D)** Representative flow cytometry results and statistical analysis of Arg-1 expression in decidual M-MDSCs and G-MDSCs from WT/INF and INF/JSI-124 mice (n = 7). **(E)** Representative flow cytometry results and statistical analysis of the intracellular level of IL-10 in decidual M-MDSCs and G-MDSCs from WT/INF and INF/JSI-124 mice (n = 7). Cells from the WT/INF mice were used for the FMO conditions. The data are presented as the mean ±SD, *p < 0.05, **p < 0.01, Student’s *t* test.

We next used JSI-124 to detect the effect of STAT3 on decidual MDSCs during *T*. *gondii* infection *in vivo* (Fig 5A). The data showed that JSI-124 did not affect pregnancy outcomes and pro-inflammatory cytokines (IFN-γ and TNF-α) levels in serum and decidua in the pregnant mice without *T*. *gondii* infection (S6 Fig). Conversely, JSI-124 obviously worsened adverse pregnancy outcomes caused by *T*. *gondii* infection and increased pro-inflammatory cytokines (IFN-γ and TNF-α) levels in serum and decidua in the presence of *T*. *gondii* infection (Figs 5B–5C and S6). Flow cytometry showed that JSI-124 effectively inhibited the activity of STAT3 in decidual MDSCs (S7C Fig) and the expression of Arg-1 in M-MDSCs and G-MDSCs from the decidua of the JSI-124-treated mice infected with *T*. *gondii* (Fig 5D). Decreased percentages of decidual IL-10^+^ M-MDSCs and IL-10^+^ G-MDSCs were also found in the mice treated with JSI-124 (Fig 5E), indicating that STAT3 plays an indispensable role in decidual MDSCs activation and pregnancy outcomes during *T*. *gondii* infection.

### Saracatinib inhibits decidual MDSCs activation and exacerbates adverse pregnancy outcomes caused by *T*. *gondii* infection

**Fig 6:**
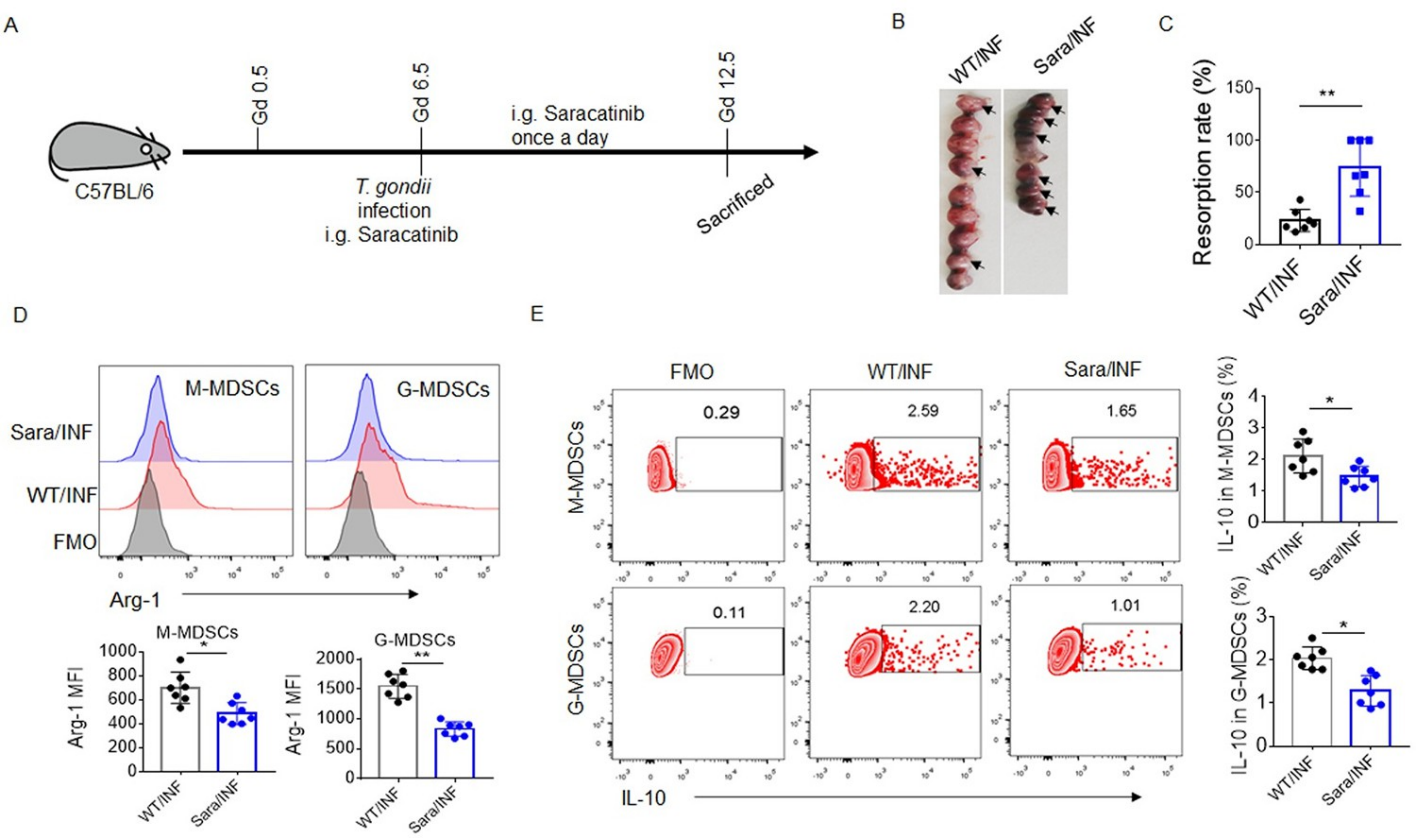
Saracatinib inhibits decidual MDSCs during *T*. *gondii* infection *in vivo*. **(A)** Schematic of the experiment. The detailed protocol is described in the *Materials and Methods*. **(B–C)** Representative picture and statistical analysis of uteri from the *T*. *gondii*-infected pregnant WT mice treated with or without saracatinib (Sara). Black arrows indicated resorption sites. Each symbol represents an individual animal, and the median is indicated (n = 7). **(D)** Representative flow cytometry results and statistical analysis of the intracellular level of p-STAT3 in decidual M-MDSCs and G-MDSCs from the infected WT mice treated with (Sara/INF) or without Sara (WT/INF) (n = 7). **(E)** Representative flow cytometry results and statistical analysis of the expression of Arg-1 in decidual M-MDSCs and G-MDSCs from WT/INF and Sara/INF mice (n = 7). **(F)** Representative flow cytometry results and statistical analysis of the intracellular level of IL-10 in decidual M-MDSCs and G-MDSCs from WT/INF and Sara/INF mice (n = 7). Cells from the WT/INF mice were used for the FMO conditions. The data are presented as the mean ± SD, *p < 0.05, **p < 0.01, Student’s *t* test.

**Fig 7:**
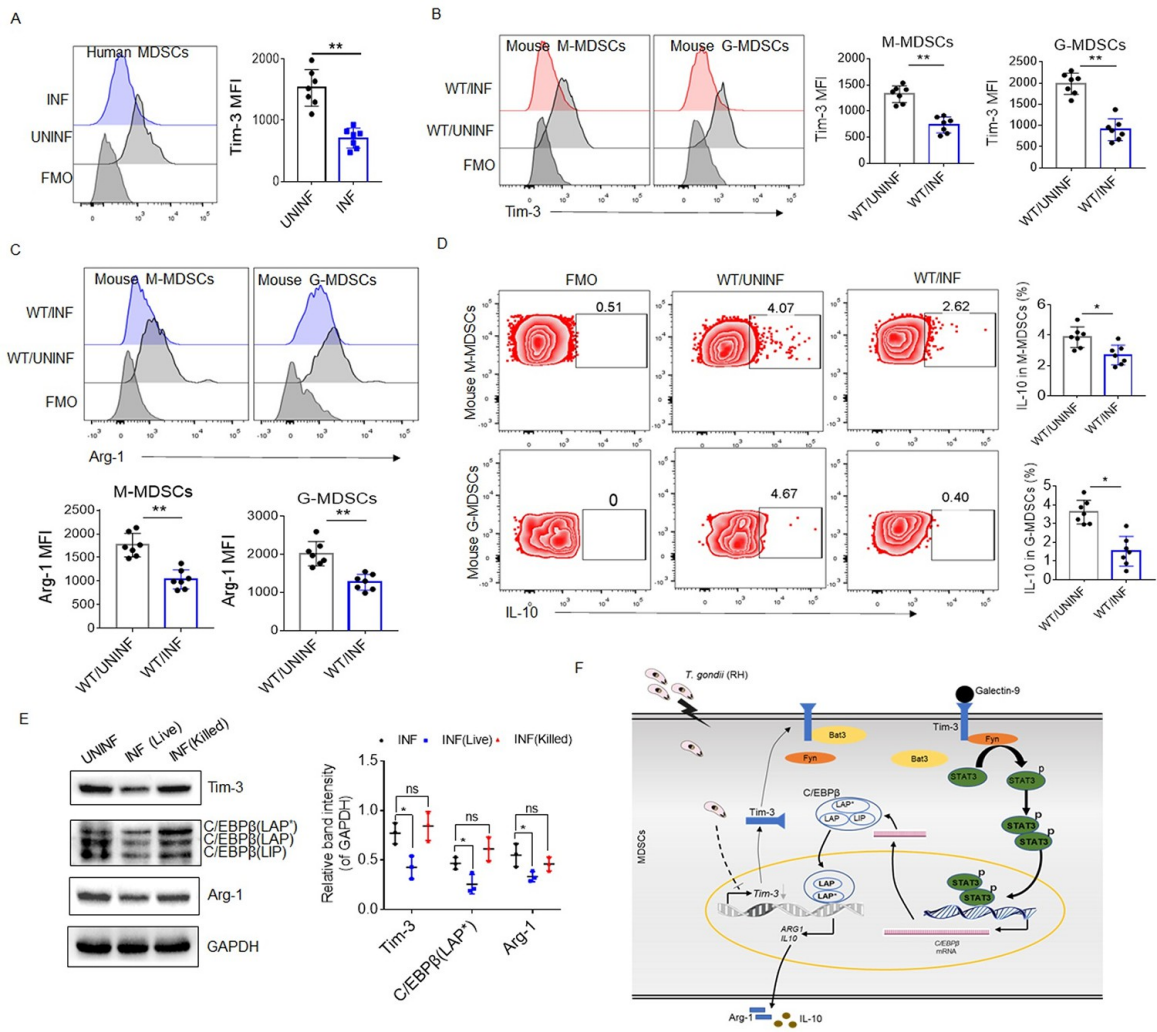
*T*. *gondii* infection inhibits Tim-3 expression in MDSCs. **(A)** Flow cytometry analyses of Tim-3 expression in human decidual MDSCs with (INF) or without (UNINF) *T*. *gondii* infection (n = 7). **(B–D)** Representative flow cytometry results and statistical analysis of the levels of Tim-3 (B), Arg-1 (C), and IL-10 (D) in decidual MDSCs from the uninfected WT (WT/UNINF) and infected WT (WT/INF) mice (n = 7). The data are presented as the mean ± SD of 7 mice, *p < 0.05, **p < 0.01, Student’s *t* test. **(E)** Western blot analyses of Tim-3, C/EBPβ, and Arg-1 expression in human decidual MDSCs infected with live *T*. *gondii* (INF (Live)), killed *T*. *gondii* by heat (IFN (Killed)), or without infection (UNINF). The quantified relative band intensity was determined by ImageJ. The results are shown as the mean ± SD of three separate experiments, One-way ANOVA, *p < 0.05. **(G)** Mechanism of how Tim-3 promotes the immunosuppressive function of MDSCs at the maternal-fetal interface. *T*. *gondii* infection inhibits the expression of Tim-3 in MDSCs. Moreover, Tim-3 signaling activated by its ligands such as galectin-9, may promote the activity of STAT3 by Fyn, then induce the expression and transcriptional activity of C/EBPβ. As a transcription factor, C/EBPβ upregulates the expression of Arg-1 and IL-10 and enhances MDSCs activation during *T*. *gondii* infection.

To further confirm the role of Fyn in decidual MDSCs activation during *T*. *gondii* infection *in vivo*, we treated pregnant mice with the Fyn inhibitor saracatinib (Fig 6A). More severe adverse pregnancy outcomes and higher IFN-γ and TNF-α levels were found in the infected pregnant mice treated with saracatinib than in the infected pregnant mice (Figs 6B–6C, S6F–S6G), however, saracatinib did not affect pregnancy outcomes and pro-inflammatory cytokines (IFN-γ and TNF-α) levels in the absence of *T*. *gondii* (S6C–S6D Fig). Furthermore, saracatinib effectively inhibited the activity of STAT3 in decidual MDSCs (Fig 6D). Lower expression of Arg-1 in decidual M-MDSCs and G-MDSCs and decreased decidual IL-10^+^ M-MDSCs and IL-10^+^ G-MDSCs were also found in the infected mice treated with saracatinib (Fig 6E and 6F), indicating that Fyn, an upstream signal molecule of STAT3, is important for decidual MDSCs activation and pregnancy outcomes during *T*. *gondii* infection. Taken together, our data indicated that the Fyn-STAT3-C/EBPβ pathway activated by Tim-3 signaling could regulate decidual MDSCs activation by increasing the expression of Arg-1 and IL-10 during *T*. *gondii* infection, which is closely related to maternal-fetal immune tolerance and pregnancy outcomes (Fig 7F).

### The expression of Tim-3 is decreased in decidual MDSCs after *T*. *gondii* infection

The level of Tim-3 expression in decidual MDSCs was explored during *T*. *gondii* infection. Decreased expression of Tim-3 in human decidual MDSCs after *T*. *gondii* infection was found by flow cytometry (Fig 7A). Consistently, the expression of Tim-3 was also downregulated in murine decidual MDSCs after *T*. *gondii* infection *in vivo* (Fig 7B). We also found that *T*. *gondii* remarkably inhibited the expression of Arg-1 and the proportion of IL-10^+^ cells in human (Fig 3A–3D) and murine (Fig 7C–7E) decidual MDSCs. In addition, western blot analyses showed that the expression of Tim-3, C/EBPβ, and Arg-1 were significantly decreased in the human decidual MDSCs after the infection with live *T*. *gondii*, whereas no alteration was found in the human decidual MDSCs treated with heat-killed *T*. *gondii* (Fig 7E), indicating that its effect may require the invasion of *T*. *gondii*. These results suggested that *T*. *gondii* invades to MDSCs and then inhibits MDSCs activation by downregulating the expression of Tim-3 (Fig 7F), which eventually contributes to the development of abnormal pregnancy outcomes.

### Adoptive transfer of decidual MDSCs from WT mice alleviates the adverse pregnancy outcomes in Tim-3KO mice

To further confirm whether the adverse pregnancy outcomes caused by *T*. *gondii* infection were partly due to the lack of Tim-3 in MDSCs, we constructed an adoptive transfer experiment that infected Tim-3KO pregnant mice were transferred with decidual MDSCs isolated from WT or Tim-3KO pregnant mice. We found the abnormal fetal rate caused by *T*. *gondii* infection was decreased after the adoptive transfer of WT decidual MDSCs compared with the transfer of Tim-3KO decidual MDSCs (S8A–S8B Fig). The decidual MDSCs from donor WT or Tim-3KO pregnant mice that reached the recipient deciduae were accounted by flow cytometry (S8C Fig). The expression of Tim-3, Arg-1, and IL-10 in decidual M-MDSCs and G-MDSCs was also increased in Tim-3KO mice transferred with WT decidual MDSCs (S8D–S8F Fig). In addition, IFN-γ level in serum and decidua was significantly lower in the mice transferred with WT decidual MDSCs than that in the mice transferred with Tim-3KO decidual MDSCs (S8G–S8H Fig).

## Discussion

*T*. *gondii* is an obligate intracellular parasite that induces a strong immune response at the maternal-fetal interface and causes abortions in humans and livestock [4]. Our previous studies have shown that *T*. *gondii* infection could result in dysfunction of decidual NK cells [23], macrophages [24], and Tregs [3]. Further studies on its regulatory mechanism showed that several immune checkpoint molecules, such as LILRB4 [2], B7-H4 [25], and Tim-3 [17], are aberrantly expressed in decidual immune cells following *T*. *gondii* infection, which impairs their regulation of the function of decidual immune cells. MDSCs are immature developing precursors of innate myeloid cells that are increased in pregnant women, indicating their possible immunosuppressive function in pregnancy [26]. These cells participate in maintaining immunological tolerance by suppressing T-cell proliferation by expressing Arg-1, iNOS, and IL-10 [11]. However, the role of MDSCs in *T*. *gondii*-induced abnormal outcomes is still unclear.

Tim-3, acting an immunosuppressive molecule, has been reported to regulate immune responses and play an important role in maternal-fetal tolerance during pregnancy. Initially, the role of Tim-3 in regulating maternal-fetal immunotolerance was studied in allogeneic pregnancy [18]. Tim-3 blockade had no effects on fetal loss and litter size in syngeneic pregnancy, but an increased rate of resorption was observed in pregnant CBA/CaJ females (mated with C57BL/6 males) challenged with Tim-3-blocking Ab [18]. Similarly, the present study revealed that there were no obvious differences in pregnancy outcomes between the pregnant WT and Tim-3KO mice, while more severe adverse pregnancy outcomes occurred in the *T*. *gondii*-infected Tim-3KO mice than in the infected WT mice. *T*. *gondii*-infected Tim-3KO pregnancy mice had higher levels of IFN-γ and TNF-α, fewer number of parasites than those of infected WT pregnancy mice, which indicated that Tim-3 deficiency might weaken the immune tolerance function and enhance killing against parasite in pregnancy mice. More severe adverse pregnancy outcomes of *T*. *gondii*-infected Tim-3KO mice may related to increased inflammation and weakened immune tolerance function. These results demonstrated that in the condition of *T*. *gondii* infection, Tim-3 may act as an anti-inflammatory molecule and play a critical role in preventing the abnormal pregnancy outcomes. Furthermore, studies have shown that Tim-3 is expressed in MDSCs and involved in MDSCs activation [18, 27]. Adverse pregnancy outcomes caused by *T*. *gondii* infection in Tim-3KO mice was alleviated following the adoptive transfer of decidual MDSCs from WT mice, indicating that abnormal pregnancy outcomes in Tim-3KO mice was related to the lack of Tim-3 in MDSCs. Thus, the aim of the present study was to explore the mechanism of Tim-3 in modulating MDSCs function during pregnancy with *T*. *gondii* infection *in vitro* and *in vivo*.

MDSCs expansion has been recognized as an important pathophysiological factor in most types of diseases, especially cancers and chronic inflammation [6]. Previous studies in mice have suggested that MDSCs significantly expand at the maternal-fetal interface, peripheral blood, and spleen throughout early to middle pregnancy [28]. In contrast, MDSCs were decreased in mice with spontaneous abortion compared with normal pregnant mice [29]. In the present research, our results showed that the proportion of M-MDSCs in the spleen, decidua, and blood from the infected pregnant Tim-3KO mice was remarkably lower than that in the infected pregnant WT mice, but no significant change was observed in the proportion of G-MDSCs. This result indicated that M-MDSCs are more strongly influenced by *T*. *gondii* infection during pregnancy than G-MDSCs.

MDSCs exert their immunosuppressive effects on the local microenvironment by expressing immunosuppressive molecules, especially Arg-1, iNOS, and IL-10 [29]. The expression of these molecules in MDSCs can indirectly reflect the activity status of MDSCs [29]. In our study, we reported that Tim-3 deficiency prominently inhibited the intracellular expression of Arg-1 and IL-10 both in M-MDSCs and G-MDSCs from the spleen, decidua, and blood of pregnant mice during *T*. *gondii* infection. Similar to the data in mice, we also found that Tim-3-blocking Ab downregulated the expression of Arg-1 and IL-10 in human decidual MDSCs with *T*. *gondii* infection *in vitro*, indicating that Tim-3 signaling facilitates the activity of MDSCs during *T*. *gondii* infection. Several studies have shown that MDSCs during pregnancy suppress T-cell proliferation to maintain maternal-fetal tolerance by expressing higher levels of immunosuppressive molecules such as Arg-1 and IL-10 [13, 28, 30]. Arg-1 could inhibit T-cell proliferation by consuming arginine and decreasing the expression of the CD3ζ chain [31], while IL-10 plays a role in the suppression of T-cell proliferation by the IL-10 receptor-mediated signaling pathway [32]. In the present study, decidual MDSCs and splenic MDSCs from the *T*. *gondii*-infected pregnant Tim-3KO mice displayed weaker suppression of T-cell proliferation than those from the infected pregnant WT mice. These results indicated that Tim-3 may enhance the immunosuppressive function of MDSCs by upregulating the expression of Arg-1 and IL-10 during *T*. *gondii* infection. Our previous studies have shown that *T*. *gondii* infection decreases the expression of Tim-3 in decidual macrophages and decidual NK cells and further destroys their roles in sustaining normal pregnancy [1, 17]. In this research, we found that Tim-3 expression in decidual MDSCs was also downregulated following *T*. *gondii* infection. Thus, the *T*. *gondii* infection*-*induced decrease in Tim-3 expression might impair the immunosuppressive function of decidual MDSCs by lowering Arg-1 and IL-10 production, which contributes to abnormal pregnancy outcomes.

Next, we explored the mechanism by which Tim-3 regulates the expression of Arg-1 and IL-10 in decidual MDSCs. Several studies have shown that the transcription factors STAT3 and C/EBPβ are critical regulators of the development and immunosuppressive function of MDSCs [33, 34]. To illustrate the molecular mechanism of Tim-3 in regulating MDSCs functions, we used αTim-3 and rGal-9 to block or activate Tim-3 signaling in human decidual MDSCs during *T*. *gondii* infection *in vitro*. Interestingly, Tim-3 signaling indeed promoted the phosphorylation of STAT3 and the expression of C/EBPβ in human decidual MDSCs. In contrast, the STAT3 inhibitor repressed the expression of C/EBPβ, Arg-1, and IL-10. Studies have shown that the Src family kinase Fyn acts as an adaptor protein binding Tim-3 and downstream molecules [21]. Here, our data revealed that Tim-3 signaling strengthened the binding capacity between Fyn and Tim-3 and Fyn and p-STAT3. Additionally, the Fyn inhibitor downregulated the expression of STAT3, C/EBPβ, Arg-1, and IL-10. C/EBPβ was reported as a transcription factor regulating the expression of Arg-1 and IL-10 [22]. In this study, the results demonstrated that Tim-3 signaling promoted the enrichment of C/EBPβ on the promotor of *Arg1* and *Il10* during *T*. *gondii* infection. On the basis of these findings, Tim-3 might upregulate Arg-1 and IL-10 in decidual MDSCs through the Fyn-STAT3-C/EBPβ signaling pathway during *T*. *gondii* infection *in vitro*. In addition, our *in vivo* experiments clarified that STAT3 and Fyn inhibitors impeded the expression of Arg-1 and IL-10 in decidual MDSCs and exacerbated adverse pregnancy outcomes in mice caused by *T*. *gondii* infection. STAT3 and Fyn inhibitors also increased IFN-γ levels in serum and decidua in the presence of *T*. *gondii* infection, which was decreased in Tim-3KO mice after the adoptive transfer of WT decidual MDSCs. Thus, systemically increased IFN-γ levels in *T*. *gondii*-infected Tim-3KO mice might result from the lack of Tim-3 in MDSCs and the weakened suppressive activity of MDSCs.

In summary, our results demonstrated that Tim-3 plays an essential role in maintaining maternal-fetal immune tolerance through the regulation of decidual MDSCs immunosuppressive activity during *T*. *gondii* infection. A signaling cascade of Fyn-STAT3-C/EBPβ is involved in Tim-3 promoting the expression of Arg-1 and IL-10 in MDSCs, which reflects the activity status of MDSCs during pregnancy with *T*. *gondii* infection. The data of the present study also demonstrated that *T*. *gondii* infection may lead to MDSCs dysfunction by inhibiting Tim-3 expression. These findings indicate a new role for Tim-3 in abnormal pregnancy caused by *T*. *gondii*, and targeting Tim-3 signaling in MDSCs may have a positive impact on pregnancy outcomes.

## Materials and methods

### Ethics statement

All procedures involving animals were conducted according to the Institutional Animal Care and Use Committee of the Model Animal Research Center. Animal experiments were approved by the Animal Ethics Committee of Binzhou Medical University.

### Mice

C57BL/6 mice (WT mice; 6- to 8-week-old females and 8- to 10-week-old males) were purchased from Pengyue Laboratory Animal Technology Co., Ltd. (Jinan, China). Tim-3 gene knockout (Tim-3KO) C57BL/6 mice were obtained from Bioray Laboratories, Inc. (Shanghai, China). Tim-3KO mice were generated by CRISPR/Cas-mediated genome engineering by Bioray Laboratories, Inc., Shanghai, China. Firstly, two gRNAs-targeting the Tim-3 gene were respectively constructed and transcribed *in vitro*. Then Cas9 and gRNA were co-injected into zygotes. The F0 and F1 mice were identified using a standard PCR-based genotyping procedure with following primers, Tim-3-S, 5’-GGCTGGCTCAAACTCACTACA-3’ and Tim-3-A, 5’- CGGACAATGATAACATGGAAA -3’, yields a 748bp product from wild-type allele. All mice were maintained in the specific pathogen-free animal house of Binzhou Medical University at 23 ± 2°C with 55 ± 5% humidity and a 12 h light/12 h dark cycle, with access to abundant sterilized water and food (Jiangsu Biological Engineering Co., Ltd., China).

### *Toxoplasma gondii* tachyzoites (RH strain)

Tachyzoites of the *T*. *gondii* RH strain were harvested from continuous cell cultures in human foreskin fibroblast (HFF) cells grown with high-glucose Dulbecco’s modified Eagle’s medium (DMEM) with 100 U/ml penicillin, 100 μg/ml streptomycin, and 10% fetal bovine serum (FBS, Gibco).

### Human samples

The study involving human samples for this study was reviewed and approved by the Ethics Committee of Binzhou Medical University (approval number 2017-016-01). All decidual tissues were collected from voluntary abortion cases in the first trimester (gestational age at 8–10 weeks) after informed consent was given. Written informed consent was obtained from all the participants. All subjects were visiting the Department of Obstetrics and Gynecology, Yantai Affiliated Hospital of Binzhou Medical University. All subjects were screened for serum hepatitis B surface antigen (HBsAg), hepatitis C virus (HCV) antibody, hepatitis D virus (HDV) antigen, HDV antibody, and HIV antibody, and positive individuals were excluded from this study. Women with acute infections, fever, severe critical illness or chronic disease were also excluded from this study.

### Mouse models

Animal experiments were conducted in strict accordance with the Guide for Care and Use of Laboratory Animals of Binzhou Medical University (license number 2017-009-09). The model of abnormal pregnancy with *T*. *gondii* infection was established according to a previous study [1] with minor modifications. Briefly, 8- to 10-week-old mice were caged together at a female-to-male ratio of 2:1. Mating was determined by the presence of a white vaginal plug. The day when the plug was detected was termed day 0.5 of gestation [gestational day (Gd) 0.5]. The pregnant mice were inoculated intraperitoneally (*i*.*p*.) with 300 tachyzoites in 200 μl of sterile PBS on Gd 6.5. The uninfected mice were treated *i*.*p*. with 200 μl sterile PBS. Fetal and maternal tissues were harvested at Gd 12.5 for analysis. Resorption sites were identified by their small size and the necrotic and hemorrhagic appearance of the embryos and the rate of reabsorption was the proportion of resorption sites in the total number of implantations.

For treatment with the STAT3 inhibitor cucurbitacin I (JSI-124; MCE), pregnant mice were inoculated *i*.*p*. with 300 tachyzoites in 200 μl of sterile PBS on Gd 6.5. Moreover, the mice were treated *i*.*p*. with 1 mg/kg JSI-124 for the next six days (every other day). For treatment with the Fyn inhibitor saracatinib (AZD0530; MCE), pregnant mice were inoculated *i*.*p*. with 300 tachyzoites in 200 μl of sterile PBS on Gd 6.5. Moreover, the mice were infused with 20 mg/kg of AZD0530 for the next six days (once a day).

### Reagents and antibodies

The reagents and antibodies used in the study are listed in S1 Table.

### DNA extraction and quantitative PCR analysis of parasite numbers

For DNA preparation, the fetoplacental tissues were lysed in 200 μl of extraction buffer (0.1 M Tris-HCl pH 9.0, 1% SDS, 0.1 M NaCl and 1 mM EDTA) and 100 μg/ml of Proteinase K (Sigma) and incubated at 50°C. DNA was purified from the tissues using the Tissue DNA Extraction Kit (Invitrogen). The DNA concentration was adjusted to 25 ng/μl for each tissue and 50 ng of DNA was used as a template. The specific primers for the *T*. *gondii* B1 gene (5′-AACGGGCGAGTAGCACCTGAGGAGA-3′ and 5′-TGGGTCTACGTCGATGGCATGACAAC-3′) were used to amplificated parasite DNA in a Bio-Rad iQ5 multicolor RT‒PCR system. A standard curve was established from *T*. *gondii* DNA extracted from 1×10^5^ parasites using 2-μl samples of serial dilutions ranging from 10,000 to 0.01 parasites. Parasite numbers were calculated by interpolation on a standard curve, with the Ct values plotted against a known concentration of parasites.

### Enzyme-linked immunosorbent assays (ELISA)

For preparation of homogenate Suspension of decidual tissue, 100 ug roughly clipped tissue was mixed with 200 μl PBS and homogenized with a homogenizer (PRO200, Pro Scientific). After 12,000 g centrifugation for 20 min at 4°C, supernatants were collected and stored at −80°C until analysis. The serum is derived from coagulated blood taken from the inner canthal venous plexus in mice.

The levels of serum were obtained and tested for TNF-α and IFN-γ levels in serum and supernatants of decidual tissue homogenates were tested by ELISA according to the manufacturer’s protocols (Enzyme-linked Biotechnology, China). Standard curves were generated using standards for each assay, and all measurements of absorbance were performed in triplicate at 450 nm. Concentrations were calculated according to standard curves and respective formulas.

### Isolation of mouse decidual mononuclear cells and MDSCs

The experimental protocol was performed as previously described [13] with modifications. Briefly, mouse placentas and uterine tissues were carefully dissected from pregnant mice at Gd 12.5 and washed twice in cold PBS. Then, the isolated tissues were cut into small pieces and digested in DMEM (BI, Israel) containing 100 U/ml hyaluronidase (Sigma, USA), 1 mg/ml collagenase type IV (Gibco, USA), and 200 U/ml DNase I (Sangon Biotech, USA) at 37°C for 50 min. The digested pieces were filtered through a 70 μm cell strainer. Mononuclear cells were collected from the white film layer after Ficoll density gradient centrifugation in mouse lymphocyte separation medium (TBD Science). Decidual and splenic MDSCs were purified using a mouse MDSCs (CD11b^+^Gr1^+^) isolation kit (Stem Cell Science) according to the manufacturer’s instructions with >95% purity ensured for experiments.

### Isolation of human decidual MDSCs

Decidual samples were immediately washed 3 times in cold PBS to remove the blood and then cut into small pieces. Tissues were digested in DMEM (BI, Israel) containing 100 U/ml hyaluronidase (Sigma, USA), 1 mg/ml collagenase type IV (Gibco, USA), and 200 U/ml DNase I (Sangon Biotech, USA) at 37°C for 60 min. The digested pieces were filtered through a 70 μm cell strainer. Mononuclear cells were collected from the white film layer after Ficoll density gradient centrifugation in human lymphocyte separation medium (TBD Science). After culture at 37°C for 1 h to remove decidual macrophages and stromal cells, human decidual MDSCs were purified using a human CD33 positive isolation kit and HLA-DR negative selection kit (both from Stem Cell Science) according to the manufacturer’s instructions. Human decidual MDSCs were prepared for subsequent experiments.

### Adoptive transfer experiment

The WT and Tim-3KO pregnant mice were sacrificed by cervical dislocation on Gd 12.5 and decidual MDSCs was isolated using the STEMCELL EasySep Mouse MDSCs (CD11b^+^Gr1^+^) Isolation Kit. Purified MDSCs were labeled with 15 μM carboxyfluorescein diacetate succinimidyl ester (CFSE) (MedChemExpress) according to the manufacturer’s instructions. The cells were resuspended in sterile PBS and diluted to 5 ×10^6^ cells per 1 ml. Tim-3KO pregnant mice on Gd 5.5 were transferred intravenously with 1 × 10^6^ decidual MDSCs isolated from WT or Tim-3KO pregnant mice. The mice of control group were injected intravenously with 200 μl sterile PBS. Then all mice were infected with 300 *T*. *gondii* tachyzoites of RH strain on Gd 6.5 and sacrificed by cervical dislocation on Gd 12.5. The pregnancy outcome was observed, and decidual mononuclear cells were isolated and analyzed by flow cytometry.

### T-cell proliferation assay

Spleen CD3^+^ T cells isolated from 4-week-old WT mice were labeled with CFSE according to the manufacturer’s instructions. MDSCs freshly isolated from the spleens or deciduae of infected WT and infected Tim-3KO mice were purified using a mouse MDSCs (CD11b^+^Gr1^+^) isolation kit (Stem Cell Science) according to the manufacturer’s instructions. 1×10^5^ MDSCs were cocultured with CD3^+^ T cells at a ratio of 1:2, 1:4 or 1:8 in the presence of 10 μg/ml anti-CD3 and 1 μg/ml anti-CD28 (BioLegend) in 96-well plates. After 3 days of incubation, the cells were resuspended in PBS for flow cytometry analysis of CFSE in CD4^+^ T and CD8^+^ T cells.

### Flow cytometric analysis

The prepared 1×10^6^ human decidual mononuclear cells were infected with *T*. *gondii* tachyzoites at a 1:2 ratio (*T*. *gondii*:cells) and/or supplemented with 10 mg/ml anti-Tim-3 mAb (Thermo Fisher Scientific) in 6-well plates. All samples were cultured in RPMI medium with 10% FBS (Gibco) and 100 IU/ml penicillin/streptomycin (Sigma-Aldrich) for 24 h at 37°C in a humidified 5% CO_2_ incubator. The prepared human and murine decidual mononuclear cell suspensions were stained with surface markers. After surface marker staining, the cells were fixed and permeabilized using CytoFix/Perm solution (Foxp3/Transcription Factor Staining Buffer Set, eBioscience) and stained with Arg-1, IL-10 or p-STAT3 antibodies according to the manufacturer’s instructions. Then, a FACSCanto II flow cytometer (BD Bioscience) was used for all flow cytometry data acquisition, and the collected data were analyzed using FlowJo analysis software (FlowJo, USA). The gating strategies are provided in S4 Fig.

### Real-time PCR (qPCR)

Total RNA was extracted from human decidual MDSCs using TRIzol reagent (Invitrogen). The RNA was transcribed to cDNA using a SuperRT cDNA Synthesis Kit (CWBIO) according to the manufacturer’s instructions. Quantitative real-time PCR (qPCR) was performed by using an UltraSYBR One Step RT‒qPCR Kit (CWBIO) in a Bio-Rad iQ5 multicolor RT‒PCR system. GAPDH mRNA expression was detected in each experimental sample as an endogenous control. The primers used for qPCR are shown in S1 Table. The expression of *ARG1* and *IL10* was calculated using the 2^−ΔΔCT^ method.

### Western blot analysis

1×10^6^ purified human decidual MDSCs were infected with *T*. *gondii* tachyzoites at a 1:2 ratio (*T*. *gondii*:cells) with or without 10 mg/ml anti-Tim-3 monoclonal antibody mAb (Thermo Fisher Scientific) and 20 μg/ml artificial recombinant galectin-9 (rGal-9). All samples were cultured in RPMI 1640 medium with 10% FBS (Gibco) and 100 IU/ml penicillin/streptomycin (Sigma-Aldrich) for 24 h at 37°C in a humidified 5% CO_2_ incubator. Cells were harvested and rinsed twice with ice-cold PBS. The cells were lysed with RIPA Lysis Buffer (Beyotime Biotechnology, China) and centrifuged at 12,000 × g for 15 min at 4°C. The protein concentrations of the extracts were measured using a bicinchoninic acid assay (Beyotime Biotechnology). Equal amounts of protein were loaded onto 10% or 12% SDS‒PAGE gels and then transferred to polyvinylidene fluoride (PVDF) membranes (Millipore). After the membranes were blocked at room temperature for 2 h in 5% nonfat dry milk in TBS-T buffer, they were incubated overnight with primary antibodies at 4°C. The membranes were washed three times for 10 min with TBS-T buffer and incubated for 1 h at room temperature with the appropriate HRP-conjugated secondary antibody. Immunoreactive bands were visualized with an enhanced chemiluminescence (ECL) detection kit (Yeasen) and analyzed using the Bio-Red ChemiDoc XRS^+^ System.

### Coimmunoprecipitation (Co-IP)

1×10^6^ purified human decidual MDSCs were infected with *T*. *gondii* tachyzoites at a 1:2 ratio (*T*. *gondii*:cells) with or without 10 mg/ml anti-Tim-3 mAb (Thermo Fisher Scientific) and 20 μg/ml rGal-9. Cells were collected and lysed with nondenatured cell lysis buffer (Beyotime Biotechnology). Cell lysates were preabsorbed with protein A/G agarose beads for 1 h at 4°C with rotation. The protein concentration was measured by bicinchoninic acid assay (Beyotime Biotechnology). Twenty percent of the whole-cell lysate was prepared for input (WCL) with 5× loading buffer boiling for 10 min at 100°C. The rest of the extracts were incubated with anti-Fyn antibody overnight with rotation at 4°C followed by incubation with protein A/G agarose beads (Santa Cruz Biotechnology) for 2 h at 4°C. The beads were washed with lysis washing buffer 3 times and denatured at 100°C for 10 min. Protein samples were subjected to Western blot analysis.

### Chromatin immunoprecipitation and qPCR (ChIP-qPCR)

Chromatin immunoprecipitation (ChIP)-qPCR was performed using a SimpleChIP Plus Enzymatic Chromatin IP Kit (Cell Signaling) according to the manufacturer’s instructions. Infected human decidual MDSCs were crosslinked with 1.5% paraformaldehyde at room temperature for 20 min. Crosslinking was stopped by adding glycine to a final concentration of 0.125 M for 5 min at room temperature. Cells were centrifuged at 500 × g for 5 min at 4°C and washed with ice-cold PBS (containing 1× protease inhibitor) 3 times. Micrococcal nuclease was used to digest DNA to a length of approximately 150–900 bp. The digested suspension was sonicated for 20 cycles of 10 sec ON and 20 sec OFF in 10 cycles on ice to lyse nuclei. For immunoprecipitation, digested chromatin preparation was incubated with C/EBPβ antibody overnight with the rotation at 4°C. Protein G agarose beads were added and incubated for 2 h at 4°C with rotation. The protein G agarose beads were washed with 1 ml of low salt at 4°C for 5 min with rotation for a total of 3 times, followed by high salt wash for 5 min with rotation. Chromatin was eluted from the antibody/protein G agarose beads for 30 min at 65°C with gentle vortexing and centrifuged at 500 × g for 1 min. Crosslinking was reversed by incubating 5 M NaCl and proteinase K for 2 h at 65°C. DNA spin columns were used to purify DNA. qPCR was performed on DNA by using an UltraSYBR One Step RT‒qPCR Kit (CWBIO) in a Bio-Rad iQ5 multicolor RT‒PCR system. The specific primers used for CHIP-qPCR are shown in S1 Table.

### Statistical analysis

Statistical analyses were performed using GraphPad Prism 7 software (GraphPad Software). Data are presented as the mean ± SD and were assessed in two-tailed Student’s *t* test or one-way ANOVA. A 95% confidence interval was considered significant and was defined as *p* < 0.05 (*p < 0.05, **p < 0.01, ***p < 0.001).

## Supporting information

S1 Fig Tim-3 deficiency enhances the levels of proinflammatory cytokines and decreases the parasite burden. **(A-B)** ELISA analysis of the levels of IFN-γ and TNF-α in serum and decidua of *T*. *gondii*-infected WT and Tim-3KO pregnancy mice (n = 5). **(C)** Parasite burden in spleen and decidua from the *T*. *gondii*-infected WT and Tim-3KO pregnancy mice (n = 8). The data are presented as the mean ± SD, Student’s *t* test, *p < 0.05.
(TIF)

S2 Fig Tim-3 deficiency has no obvious effects on pregnancy outcomes in mice without *T. gondii* infection. **(A)** Representative picture and statistical analysis of uteri from pregnant WT and Tim-3KO mice (n = 15). **(B)** Representative flow cytometry results and statistical analysis of M-MDSCs and G-MDSCs in spleens, blood, and deciduae from the WT and Tim-3KO mice (n = 7). **(C-D)** ELISA analysis of the levels of IFN-γ and TNF-α in serum and decidua of pregnant WT and Tim-3KO mice without *T*. *gondii* infection (n = 5). The data are presented as the mean ± SD, Student’s *t* test.
(TIF)

S3 Fig Tim-3 deficiency inhibits blood MDSCs activation during *T. gondii* infection. **(A)** Representative flow cytometry results and statistical analysis of M-MDSCs and G-MDSCs in blood from infected WT (WT/INF) and Tim-3KO mice (Tim-3KO/INF) (n = 7). **(B)** Representative flow cytometry results and statistical analysis of the expression of Arg-1 in M-MDSCs and G-MDSCs from the blood of the WT/INF group and Tim-3KO/INF group (n = 7). **(C)** Representative flow cytometry results and statistical analysis of the intracellular level of IL-10 in M-MDSCs and G-MDSCs from the blood of the WT/INF and Tim-3KO/INF (n = 7). Cells from the WT/INF group were used for all the FMO conditions. The data are presented as the mean ±SDs, *p < 0.05, **p < 0.01, Student’s *t* test.
(TIF)

S4 Fig Representative FACS gating scheme for immune cell analyses in this study. **(A-B)** Gating strategy for mouse M-MDSCs and G-MDSCs. After eliminating double cells by FCS-W and SSC-W, we gated on CD11b^+^ cells for further analyses following initial gating on CD45^+^ cells. Then, M-MDSCs were gated by Ly-6G^-^Ly6C^hi^, and G-MDSCs were gated by Ly-6G^+^Ly6C^low^. **(C)** Gating strategy for human decidual MDSCs. After eliminating double cells by FCS-W and SSC-W and dead cells by 7-AAD, we gated on CD33^+^HLA-DR^-^ cells for further analyses following initial gating on live CD45^+^ cells.
(TIF)

S5 Fig Western blot analysis of C/EBPβ and Arg-1 expression in human decidual MDSCs treated with αTim-3 and/or *T. gondii* infection. The quantified relative band intensity was determined by ImageJ. The results are shown as the mean ±SD of three separate experiments, One-way ANOVA, *p < 0.05.
(TIF)

S6 Fig The effects of JSI-124 and saracatinib on pregnancy outcomes, parasite burden and pro-inflammatory cytokines. **(A-B)** Representative picture and statistical analysis of uteri from untreated, JSI-124 treated, and saracatinib treated pregnant WT mice without *T*. *gondii* infection (n = 7). **(C-D)** ELISA analysis of the levels of TNF-α and IFN-γ in serum and deciduae of untreated (WT/INF), JSI-124 treated (JSI-124/UNINF), and saracatinib treated (Sara/UNINF) pregnant WT mice without *T*. *gondii* infection (n = 5). **(E)** Parasite burden in spleen and decidua from groups of *T*. *gondii* infection (WT/INF), JSI-124 treatment plus infection (JSI-124/INF), and saracatinib treatment plus infection (Sara/INF) (n = 8). **(F-G)** ELISA analysis of the levels of TNF-α and IFN-γ in serum and decidua from groups of INF, JSI-124/INF, and Sara/INF (n = 5). The data are presented as the mean ±SD, One-way ANOVA, *p < 0.05.
(TIF)

S7 Fig Flow cytometry analysis of the intracellular level of p-STAT3. **(A)** Representative flow cytometry results and statistical analysis of the intracellular level of p-STAT3 in infected human decidual MDSCs after αTim-3 treatment. **(B)** Representative flow cytometry results and statistical analysis of the intracellular level of p-STAT3 in decidual MDSCs from the infected WT and Tim-3KO mice. **(C)** Representative flow cytometry results and statistical analysis of the intracellular level of p-STAT3 in decidual MDSCs from the infected WT mice treated with or without JSI-124. The data are presented as the mean ± SD, *p < 0.05, **p < 0.01, Student’s *t* test.
(TIF)

S8 Fig Adoptive transfer of decidual MDSCs from WT mice alleviates the adverse pregnancy outcomes caused by *T. gondii* in pregnant Tim-3KO mice. **(A)** Representative pictures and statistical analysis of uteri from the *T*. *gondii*-infected pregnant Tim-3KO mice transferred with decidual MDSCs from Tim-3KO mice or wild-type mice. Black arrows indicated resorption sites. Each symbol represents an individual animal, and the median is indicated (n = 6). **(B)** Representative flow cytometry results and statistical analysis of decidual CFSE^+^CD11b^+^ cells (n = 6). **(C)** Flow cytometry analysis of Tim-3 expression in decidual MDSCs (n = 6). **(D)** Representative flow cytometry results and statistical analysis of the expression of Arg-1 in decidual M-MDSCs and G-MDSCs (n = 6). **(E-F)** Representative flow cytometry results and statistical analysis of the intracellular level of IL-10 in decidual M-MDSCs and G-MDSCs (n = 6). **(G-H)** ELISA analysis of the levels of IFN-γ in serum and decidua (n = 6). The results are shown as the mean ±SD of three separate experiments, One-way ANOVA, *p < 0.05, **p < 0.01.
(TIF)

S1 Table Reagents and antibodies used in this study. (XLSX)
