# Supplementary figures and images for: Tim-3 regulates the immunosuppressive function of decidual MDSCs via the Fyn-STAT3-C/EBPβ pathway during Toxoplasma gondii infection
Source: PLoS Pathog. 2023 Apr 14;19(4):e1011329. doi: 10.1371/journal.ppat.1011329 (PMC10132691; doi:10.1371/journal.ppat.1011329)

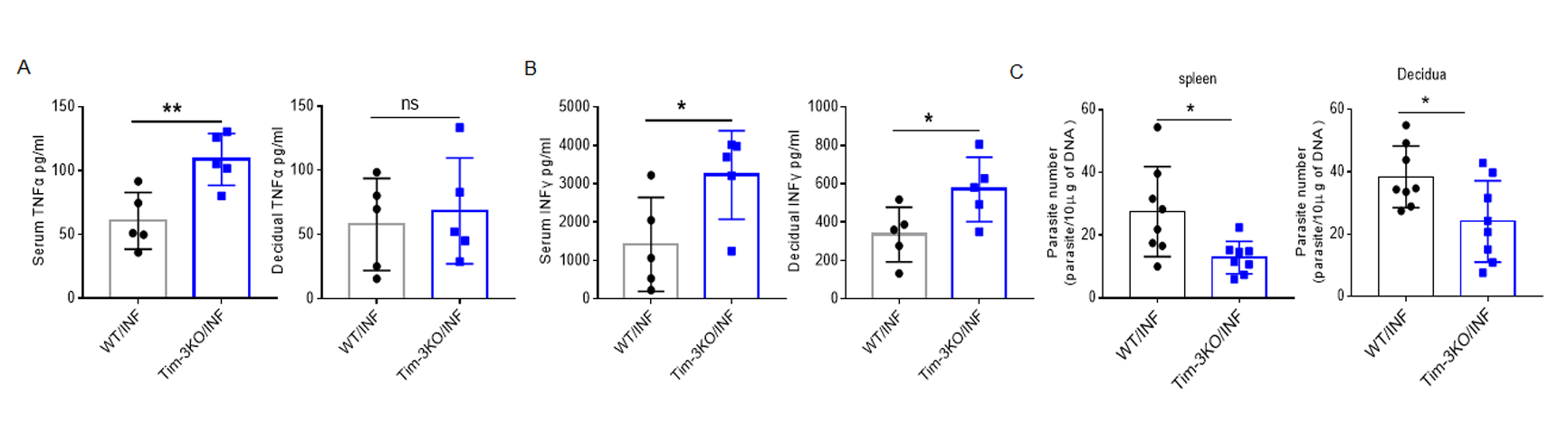

Supplement: S1 Fig — (A-B) ELISA analysis of the levels of IFN-γ and TNF-α in serum and decidua of T. gondii-infected WT and Tim-3KO pregnancy mice (n = 5). (C) Parasite burden in spleen and decidua from the T. gondii-infected WT and Tim-3KO pregnancy mice (n = 8). The data are presented as the mean ± SD, Student’s t test, *p < 0.05. (TIF) [file ppat.1011329.s001.tif]

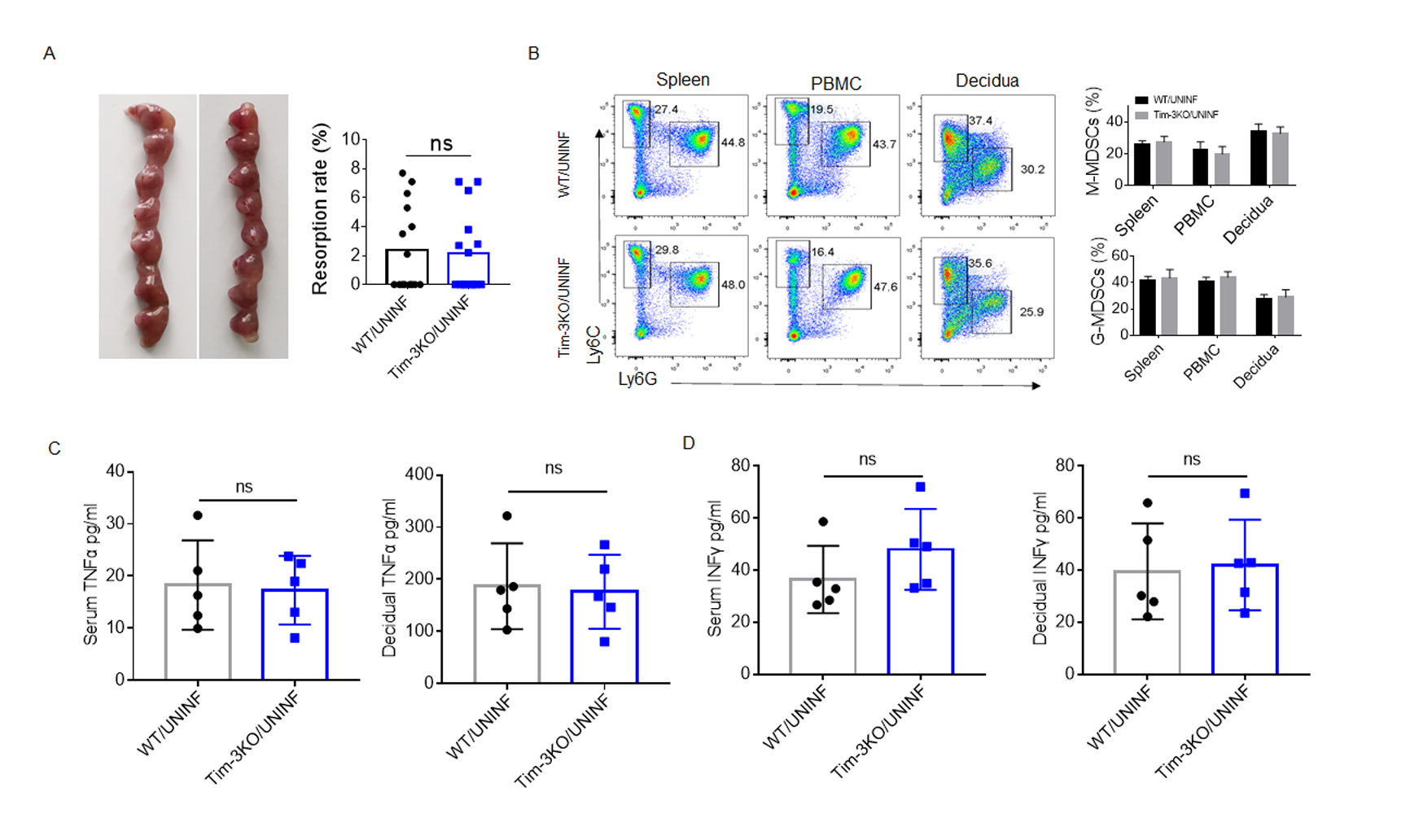

Supplement: S2 Fig — (A) Representative picture and statistical analysis of uteri from pregnant WT and Tim-3KO mice (n = 15). (B) Representative flow cytometry results and statistical analysis of M-MDSCs and G-MDSCs in spleens, blood, and deciduae from the WT and Tim-3KO mice (n = 7). (C-D) ELISA analysis of the levels of IFN-γ and TNF-α in serum and decidua of pregnant WT and Tim-3KO mice without T. gondii infection (n = 5). The data are presented as the mean ± SD, Student’s t test. (TIF) [file ppat.1011329.s002.tif]

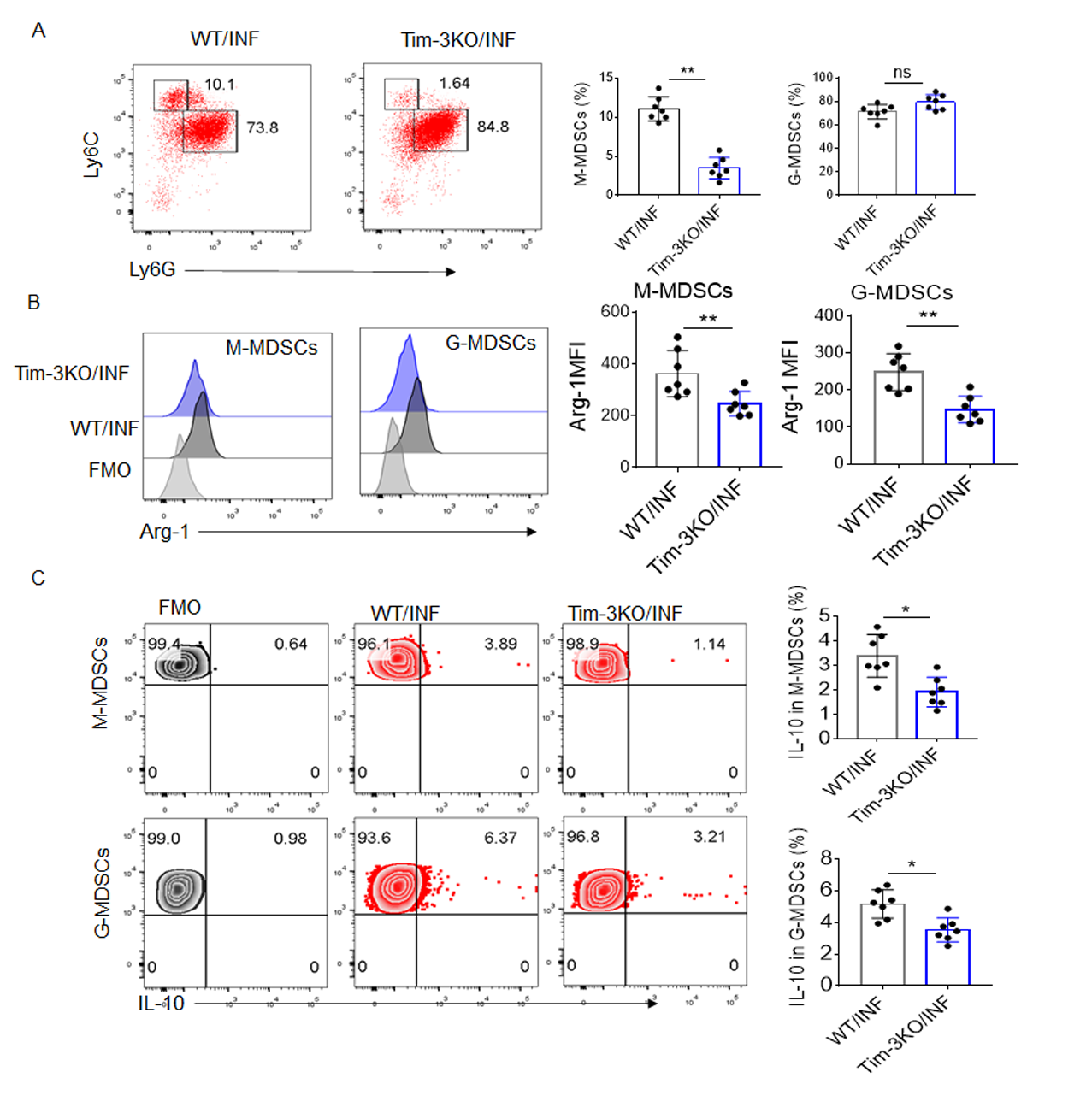

Supplement: S3 Fig — (A) Representative flow cytometry results and statistical analysis of M-MDSCs and G-MDSCs in blood from infected WT (WT/INF) and Tim-3KO mice (Tim-3KO/INF) (n = 7). (B) Representative flow cytometry results and statistical analysis of the expression of Arg-1 in M-MDSCs and G-MDSCs from the blood of the WT/INF group and Tim-3KO/INF group (n = 7). (C) Representative flow cytometry results and statistical analysis of the intracellular level of IL-10 in M-MDSCs and G-MDSCs from the blood of the WT/INF and Tim-3KO/INF (n = 7). Cells from the WT/INF group were used for all the FMO conditions. The data are presented as the mean ±SDs, *p < 0.05, **p < 0.01, Student’s t test. (TIF) [file ppat.1011329.s003.tif]

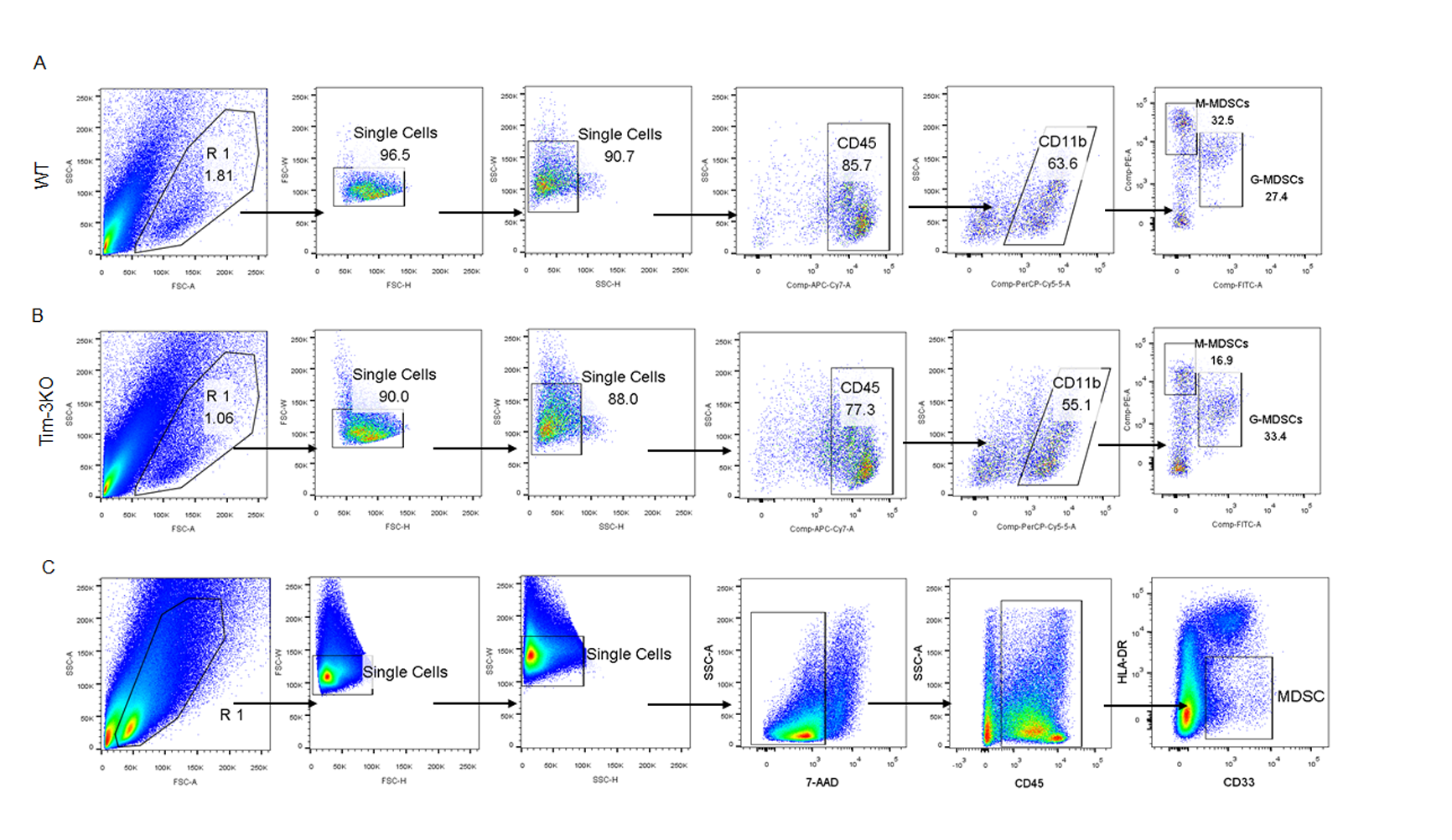

Supplement: S4 Fig — (A-B) Gating strategy for mouse M-MDSCs and G-MDSCs. After eliminating double cells by FCS-W and SSC-W, we gated on CD11b+ cells for further analyses following initial gating on CD45+ cells. Then, M-MDSCs were gated by Ly-6G-Ly6Chi, and G-MDSCs were gated by Ly-6G+Ly6Clow. (C) Gating strategy for human decidual MDSCs. After eliminating double cells by FCS-W and SSC-W and dead cells by 7-AAD, we gated on CD33+HLA-DR- cells for further analyses following initial gating on live CD45+ cells. (TIF) [file ppat.1011329.s004.tif]

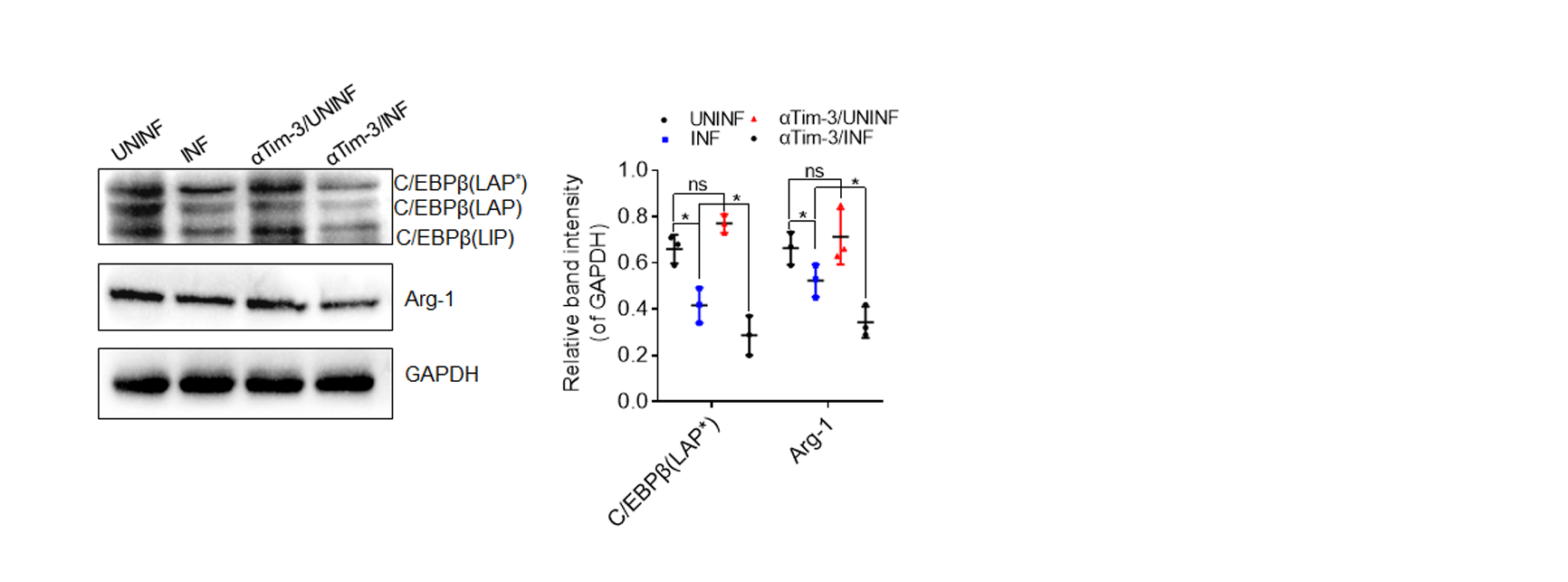

Supplement: S5 Fig — The quantified relative band intensity was determined by ImageJ. The results are shown as the mean ±SD of three separate experiments, One-way ANOVA, *p < 0.05. (TIF) [file ppat.1011329.s005.tif]

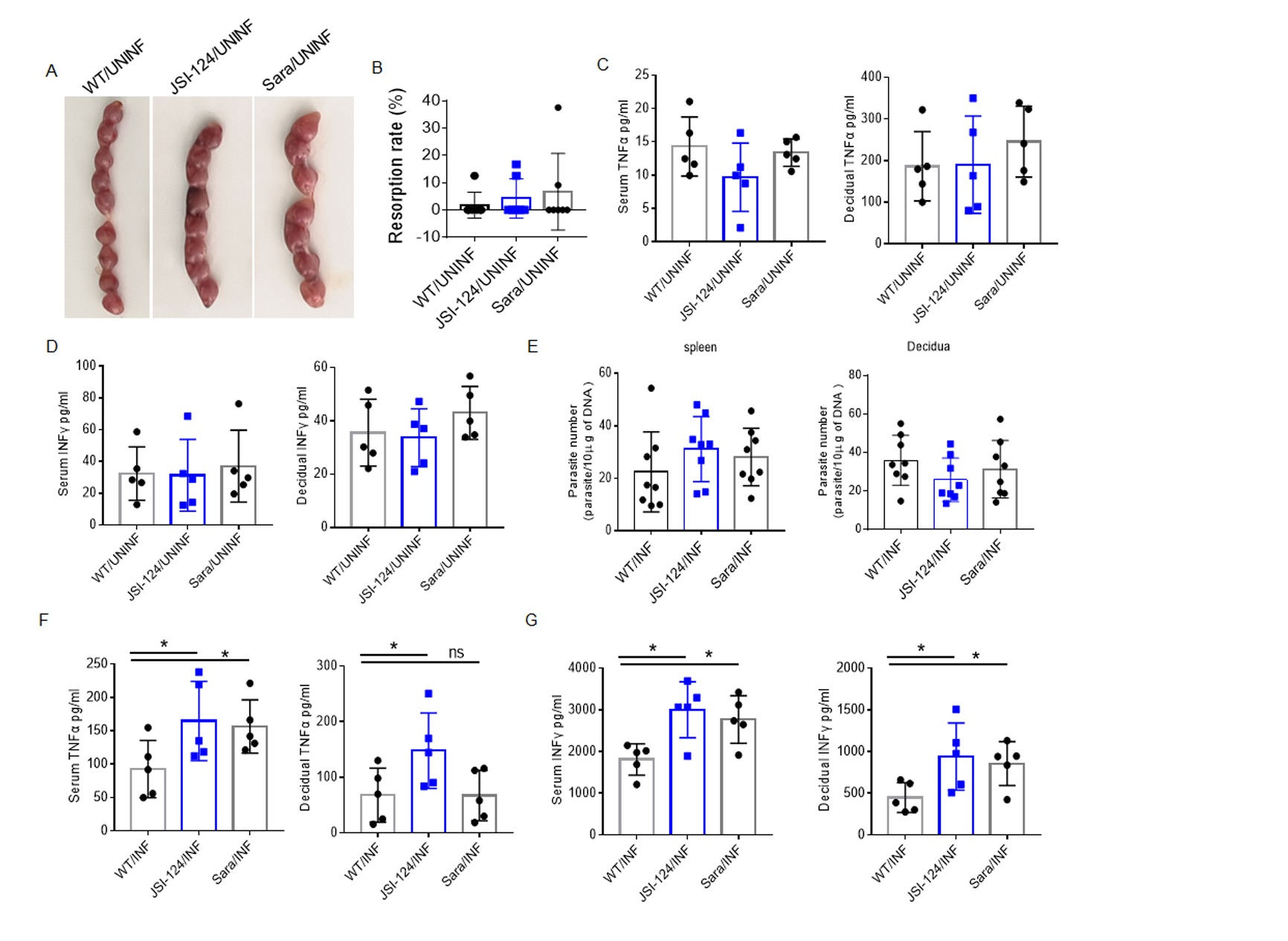

Supplement: S6 Fig — (A-B) Representative picture and statistical analysis of uteri from untreated, JSI-124 treated, and saracatinib treated pregnant WT mice without T. gondii infection (n = 7). (C-D) ELISA analysis of the levels of TNF-α and IFN-γ in serum and deciduae of untreated (WT/INF), JSI-124 treated (JSI-124/UNINF), and saracatinib treated (Sara/UNINF) pregnant WT mice without T. gondii infection (n = 5). (E) Parasite burden in spleen and decidua from groups of T. gondii infection (WT/INF), JSI-124 treatment plus infection (JSI-124/INF), and saracatinib treatment plus infection (Sara/INF) (n = 8). (F-G) ELISA analysis of the levels of TNF-α and IFN-γ in serum and decidua from groups of INF, JSI-124/INF, and Sara/INF (n = 5). The data are presented as the mean ±SD, One-way ANOVA, *p < 0.05. (TIF) [file ppat.1011329.s006.tif]

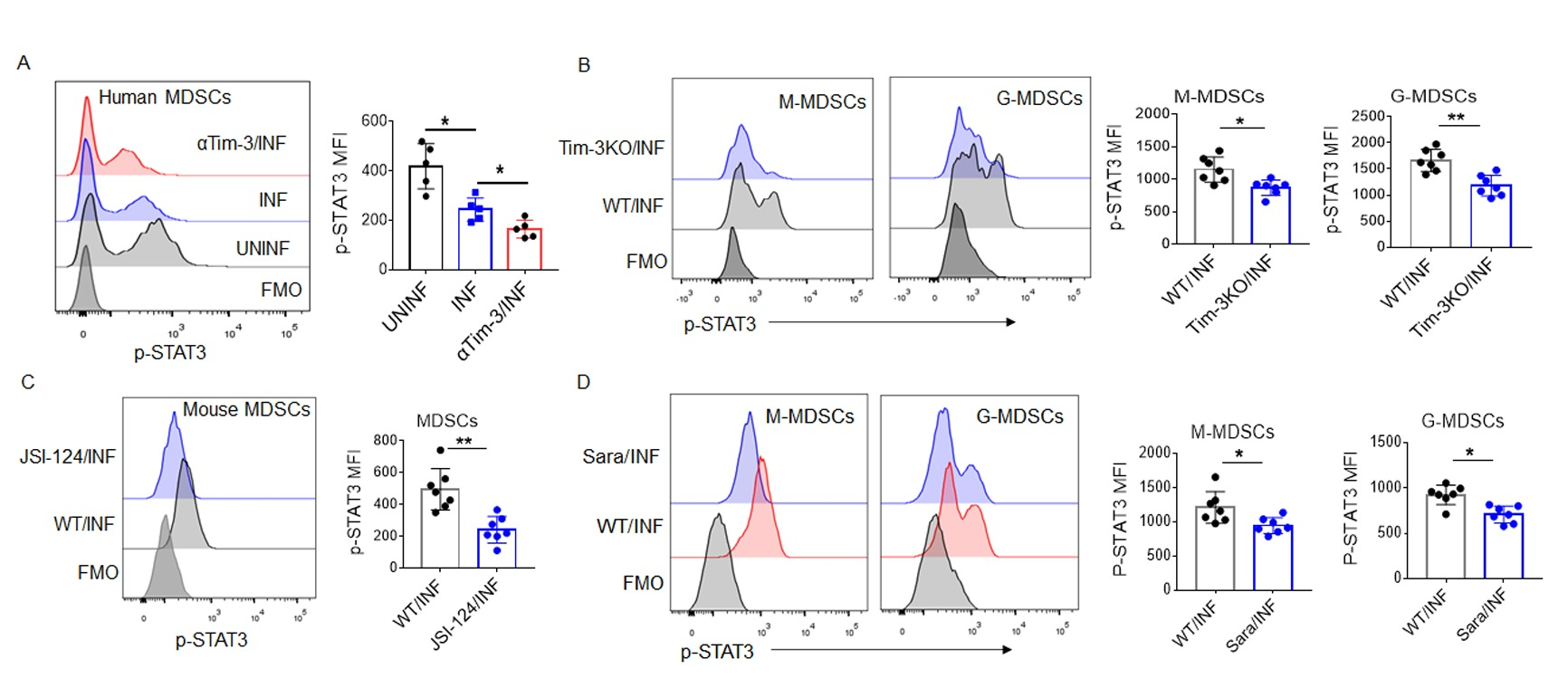

Supplement: S7 Fig — (A) Representative flow cytometry results and statistical analysis of the intracellular level of p-STAT3 in infected human decidual MDSCs after αTim-3 treatment. (B) Representative flow cytometry results and statistical analysis of the intracellular level of p-STAT3 in decidual MDSCs from the infected WT and Tim-3KO mice. (C) Representative flow cytometry results and statistical analysis of the intracellular level of p-STAT3 in decidual MDSCs from the infected WT mice treated with or without JSI-124. The data are presented as the mean ± SD, *p < 0.05, **p < 0.01, Student’s t test. (TIF) [file ppat.1011329.s007.tif]

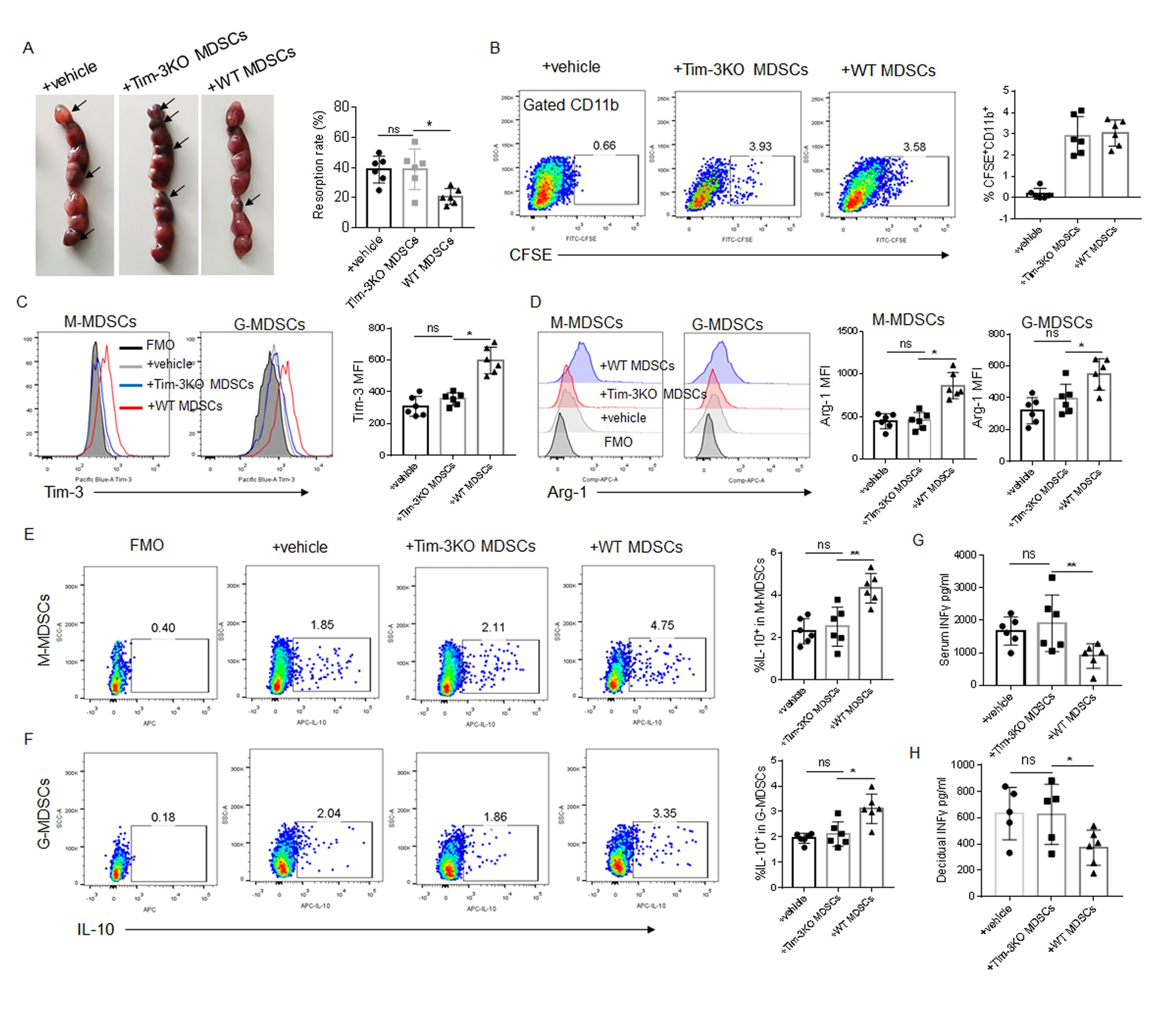

Supplement: S8 Fig — (A) Representative pictures and statistical analysis of uteri from the T. gondii-infected pregnant Tim-3KO mice transferred with decidual MDSCs from Tim-3KO mice or wild-type mice. Black arrows indicated resorption sites. Each symbol represents an individual animal, and the median is indicated (n = 6). (B) Representative flow cytometry results and statistical analysis of decidual CFSE+CD11b+ cells (n = 6). (C) Flow cytometry analysis of Tim-3 expression in decidual MDSCs (n = 6). (D) Representative flow cytometry results and statistical analysis of the expression of Arg-1 in decidual M-MDSCs and G-MDSCs (n = 6). (E-F) Representative flow cytometry results and statistical analysis of the intracellular level of IL-10 in decidual M-MDSCs and G-MDSCs (n = 6). (G-H) ELISA analysis of the levels of IFN-γ in serum and decidua (n = 6). The results are shown as the mean ±SD of three separate experiments, One-way ANOVA, *p < 0.05, **p < 0.01. (TIF) [file ppat.1011329.s008.tif]
